# Impacts of gene duplication in the evolution of symbiotic root nodule symbiosis in legumes

**DOI:** 10.3389/fpls.2026.1784647

**Published:** 2026-05-12

**Authors:** Hyun-oh Lee, Andrew D. Farmer, Jamie A. O’Rourke, Jeffrey J. Doyle, Steven B. Cannon

**Affiliations:** 1 Oak Ridge Institute for Science and Education (ORISE) Fellow, U.S. Department of Agriculture–Agricultural Research Service (USDA-ARS), Corn Insects and Crop Genetics Research Unit, Ames, IA, United States; 2 National Center for Genome Resources, Santa Fe, NM, United States; 3 Corn Insects and Crop Genetics Research Unit, USDA-Agricultural Research Service, Ames, IA, United States; 4 Section of Plant Biology and Section of Plant Breeding & Genetics, School of Integrative Plant Science, Cornell University, Ithaca, NY, United States

**Keywords:** expression divergence, gene duplication, legume (nodules), legume evolution, nitrogen-fixing nodulation clade, root nodule symbiosis, tandem duplication

## Abstract

Root nodule symbiosis (RNS) is found in approximately 16–18 widely-separated lineages within the “nitrogen-fixing nodulation clade (NFNC)”. Although modeling of trait gain and loss across approximately 13,000 species within the rosid group indicates multiple gains and losses, there is no consensus about whether RNS had a single or multiple origins; and our understanding is fragmentary regarding the molecular mechanisms underlying those changes. Evolution of a new organ and functions involves many thousands of genes; but the evolutionary histories for many of these genes may be uninformative regarding RNS evolution. A portion of the genes, however, are likely to be derived from prior gene duplications and to have acquired new functions or to have come under new regulatory patterns. Whole genome duplications (WGDs) could conceivably enable the necessary neo- or sub-functionalization for new roles in the nodule. All species that exhibit RNS share a history of several ancient WGDs; but the last such common WGD for these species was the “gamma” paleohexaploidy that occurred early in the core eudicot lineage, ~120 Mya. This presents a puzzle: If legume RNS within the NFNC only arose in the Late Cretaceous, several tens of millions of years after the gamma event, what explains the long, seemingly quiescent interval and the many eudicot lineages without RNS? This study focuses on a collection of gene superfamilies with additional independent WGDs that appear to have occurred in the interim period, after the gamma triplication and prior to the evolution of RNS, identifying several that are both essential for RNS and that show evidence of critical roles of both ancient WGDs and more recent local duplications.

## Introduction

Symbiotic nitrogen fixation (SNF) - and more specifically, root nodule symbiosis (RNS), occurring in the specialized nodule generally on root tissue, is found in many legume species and in other lineages scattered across three orders sister to the legumes (Rosales, Fagales, Cucurbitales). Taking RNS “origin” to mean assembly of all of the components necessary for a fully-functioning symbiosis occurring in structures we call “nodules,” the mechanistic history and timing of RNS evolution remains largely unknown. As elaborated in Doyle et al. (2025), two broad scenarios remain in play: a single assembly, in which conserved (deeply homologous) modular components were brought together early, near the “nitrogen-fixing nodulation clade” (NFNC) common ancestor, followed by selective losses or lineage-specific modifications, as proposed in phylogenomic and phylotranscriptomic analyses supporting a predominant single-origin model (Griesmann et al., 2018; Van Velzen et al., 2018; Libourel et al., 2023); or multiple assembly, in which the same essential components were progressively assembled over a longer time period, potentially with different components in different lineages, and with final assembly of RNS (i.e., symbiotic bacteria housed in nodules) occurring independently after divergence of NFNC lineages. Both the “single” and “multiple” scenarios could be compatible with the observed pattern of presence and absence of RNS across the NFNC (Soltis et al., 1995). In both scenarios, it is likely that essential functionalities were present prior to the radiation of the NFNC (Soltis et al., 1995), including preexisting arbuscular mycorrhizal signaling capacity, co-opted for use in the nodule (Kundu et al., 2025). Based on the taxonomic distributions of RNS and modeling of potential gains and losses of the nodule across the “nitrogen fixing nodulation clade” (NFNC), Kates et al. (2024) conclude that RNS likely evolved semi-independently numerous times (approximately 16-18) and was also lost independently in some clades (approximately 10). However, the Kates et al. (2024) modeling only assessed the observed presence or absence of RNS in extant species, rather than the molecular mechanisms that might have been responsible for gains or losses, or the timing and nature of RNS origin(s). A recent phylogenomic analysis of RNS in the NFNC presents a model of “a single foundational origin of the RNS regulatory network components during the γ paleohexaploidy occurring at ∼110 mya, followed by its initial assembly in the NFC and repeated losses in nonnodulating lineages” (Liu et al., 2026). The present study follows a similar phylogenomic strategy to examine and characterize evolutionary mechanisms seen in a collection of gene superfamilies with RNS involvement. We do not directly address the question of “single” vs. “multiple” assemblies of RNS, as we lack access to the key evolutionary artifacts: the incipient nodule structure(s) near the time of their origin(s). Rather, we focus on the timing and nature of gene duplications in the evolution of RNS. The basic hypothesis is that gene duplications of several types were important in the evolution of RNS. The evidence to be examined is in the form of gene families, genomic synteny, and gene expression patterns, with the timing of duplications being inferred relative to speciation events.

Given that a complex organ and symbiotic syndrome (the nodule and RNS) has arisen within a derived clade within the Fabidae [Fabales, Fagales, Cucurbitales, Rosales, abbreviated “FaFaCuRo” by Parniske (2018)], it is natural to ask what evolutionary events may have led to both the potentially repeated acquisitions and losses of the new structure and symbiotic capacity (regardless of single or multiple origin). Plants that are able to engage in RNS acquire substantial benefits. These advantages frequently translate into increased survival, reproduction, and competitiveness; but there is also an energetic cost to plants with RNS as they supply fixed carbon to the bacterial symbionts (Vitousek et al., 2013).

As the nodule is a complex organ, involving expression of many thousands of genes, numerous developmental modules undoubtedly comprise the nodule. These modules perform varied roles such as the cell cycle, meristematic regulation, cell growth and development, defense response, nutrient sensing and transport, etc. These roles also include more specialized functions such as sensing and communication with the symbiotic partner (rhizobial or actinorhizal bacteria), growth and management of specialized structures such as the infection thread, and development and regulation of the plant-derived peri-bacterial membrane that surrounds the symbiotic bacteria in most nodules (Soyano and Hayashi, 2014; Parniske, 2018).

The common symbiosis signaling pathway (CSSP) is central to nodule development and is activated by rhizobial nod factors and mycorrhizal signals (Oldroyd, 2013; Roy et al., 2020). This pathway includes important parts like Ca^2+^ signaling and the transcription factor Nodule Inception (NIN), which works as a main controller of nodulation (Shen and Feng, 2024; Wang et al., 2025). Cathebras et al. (2026) identified a key cis-regulatory element within the NIN promoter called the predisposition associated cis-regulatory element (PACE). PACE is exclusively found among members of the FaFaCuRo clade, regardless of nodulation phenotype (Cathebras et al., 2026). This molecular conservation supports the hypothesis that critical genetic components of nodulation were present in the FaFaCuRo clade’s shared ancestor. Despite the subsequent diversification or cessation of nodulation phenotypes across diverse lineages, the presence of this conserved cis-regulatory element is congruent with a hypothesis of a singular evolutionary origin for RNS genes, facilitated by molecular pre-adaptation. In addition, Nosaki et al. (2026) showed that the broad DNA-binding capacity of NIN is inherited from NLP (Nodule Inception-Like Protein) transcription factors, providing the mechanistic basis for its role as a master regulator. Yu et al. (2024) demonstrated that conserved cis-elements within the promoter of NAD1 enable direct NIN-dependent regulation, thereby linking nodule organogenesis with the suppression of plant defense responses.

**Figure 1:**
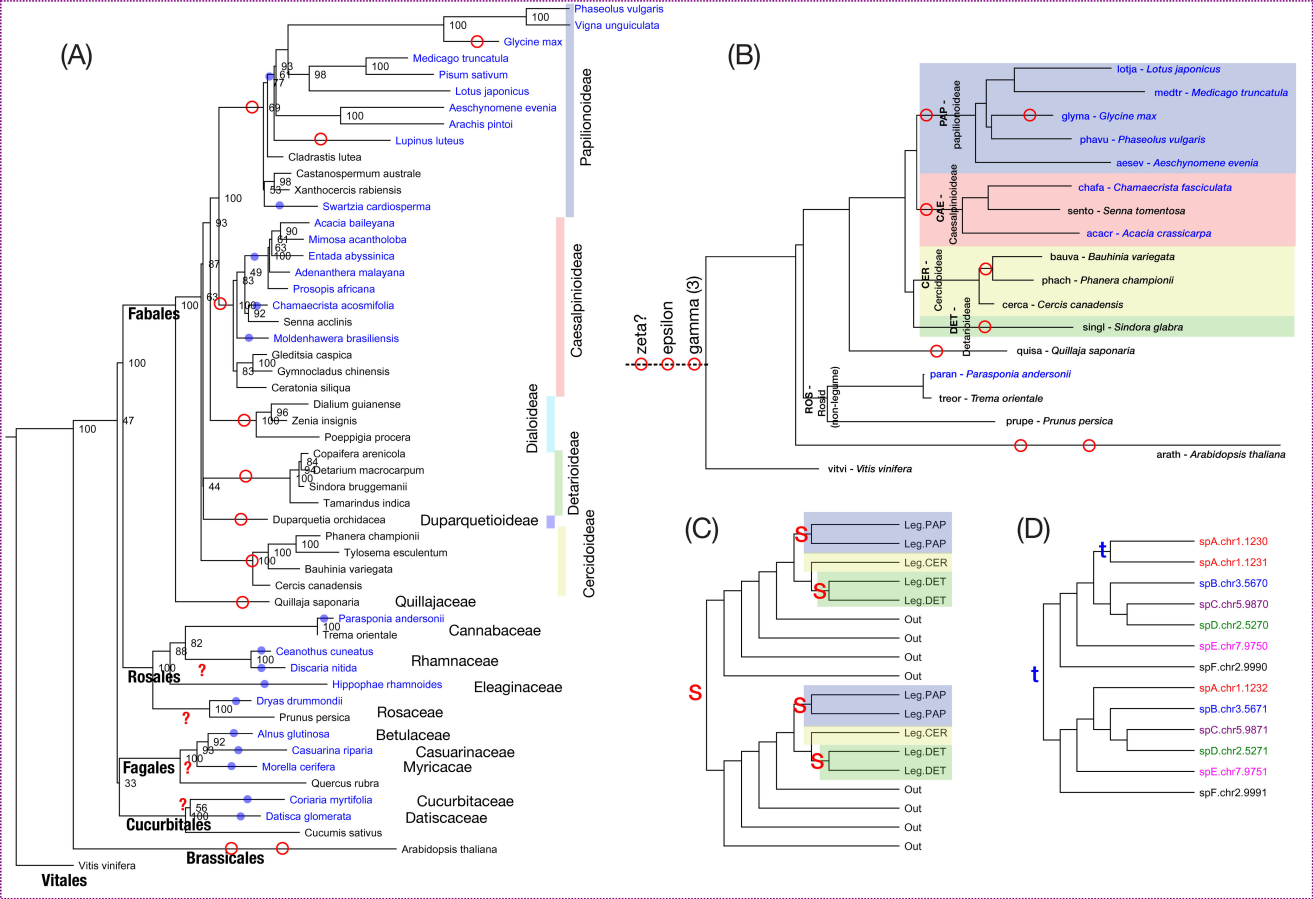
Phylogeny of species used in this study. **(A)** Species phylogeny based on matK genes for selected species in the four orders in which RNS is found. Blue text indicates observed root nodulation. Blue dots indicate approximate inferred location of gain of the RNS trait, per Kates et al. (2024). **(B)** Species phylogeny for the species used for gene family and synteny analyses in this study. Each species is preceded with a five-letter abbreviation that is used in some analyses: three letters from the genus and two of the species, e.g., “lotja” for *Lotus japonicus*. The phylogeny was derived from 3071 low-copy gene trees, using Astral-pro3. Legume subfamilies are indicated with colored blocks: Papilionoideae in blue, Caesalpinioideae in red, Cercidoideae in yellow, Detarioideae in green. Legume subfamilies not represented: Dialoideae and Duparquetioideae. Non-legume species, in order of distance from the legumes, are *Quillaja saponaria* (from the closest non-legume family, Quillajaceae); *Parasponia andersonii* (the only known non-legume lineage that nodulates with rhizobia) and its non-nodulating sister genus *Trema orientale* (two species in the Cannabaceae); *Prunus persica* (a non-nodulating species within Rosaceae); *Arabidopsis thaliana* (Brassicales, Rosids); *Vitis vinifera* (Vitales, early-diverging within the Rosids). Approximate locations of polypolyploidy are indicated with red circles. Early named polyploid events are indicated (without scale) at the root: first the known gamma triplication, preceded by the hypothesized early epsilon and zeta events. **(C)** Cartoon phylogeny showing the inference of WGD events, also termed segmental duplications (red “S”), on the basis of clades with duplicated membership; in the case of the oldest WGD shown, consisting of clades each containing legume and non-legume (outgroup) species. **(D)** Cartoon phylogeny showing the inference of local or tandem gene duplication events (blue “t”), on the basis of duplication in phylogenetic clades, in which paralogous genes are also genomically near one another (same chromosome and nearby ordinal position).

It would seem parsimonious to posit that key predisposing innovations occurred near the origin of the FaFaCuRo clade (Soltis et al., 1995), roughly 92–118 Mya (**Figure 1**) (Bell et al., 2010; Magallón et al., 2015; Parniske, 2018). However, Werner et al. (2014) suggest that a precursor trait may have arisen earlier, prior to the NFNC ancestor, and further introduce the concept of “hidden states”—unobserved enabling conditions that could facilitate both the origin and stabilization of nodulation. Nevertheless, genes that are both required for RNS and show nodule-biased expression as defined by our tissue-based RNA-seq classification (nodule/root/shoot) must have undergone changes in structure or regulation that enabled their current roles in nodulation. A gene required for RNS must either (1) be novel to the organism (Jiang et al., 2022), or (2) have existed in another form or with different regulation and had some other role previously, or (3) have duplicated such that one of the duplicates could take on a new role in the new structure. Note that these “required by” and “specific to” conditions may apply to some small subset of genes expressed in the nodule; many other genes may either be incidentally expressed in the nodule but not be essential for nodule function, or may be expressed in the nodule but also in other tissues (rather than specific to the nodule). Examples of genes that might be present in the nodule but also have essential roles in other tissues include “housekeeping genes,” which are varied and numerous (Joshi et al., 2022).

Considering the set of genes that are required for RNS and specific to the nodule, the three types of evolutionary trajectory for a gene may be labeled (1) novel, (2) neofunctionalized without duplication, or (3) neofunctionalized after duplication. Of these trajectories, the third seems intuitively the most plausible, since (1) the origin of new genes requires that a gene and gene product acquire the necessary function *sui generis*; and (2) neofunctionalization without duplication implies a shift from a prior function to the new function in the nodule; whereas (3) gene duplication is rife in plant evolution and it affords the evolution of new functions without disruption of the prior roles continued by the sibling paralogs. Note that these conditions pertain to genes that are operationally defined as nodule-specific based on expression criteria rather than to genes that may have related or even identical functions in other tissues or conditions. For example, it is clear that much of the machinery of nodulation signaling has been recruited directly from mycorrhizal signaling (Parniske, 2008; Markmann and Parniske, 2009; Soyano et al., 2021; Wang et al., 2022; Zhang et al., 2024a); but the two developmental processes and structures are also strikingly distinct. Genes from the vesicle-associated membrane protein 72 family (VAMP72) are required for both membrane invagination in arbuscular development and symbiosome formation (Sogawa et al., 2019; Wang et al., 2022); but the receptor proteins *LYK3/NFR1* and *NFP/NFR5* function specifically in recognition of nod factors (Indrasumunar et al., 2011; Girardin et al., 2019; Wong et al., 2019; Wang et al., 2022).

Thus, the duplication histories of genes involved in RNS are attractive evolutionary features in which to look for events that may have contributed to the evolution of RNS. The relevant duplication landscape includes one or more old WGDs from early in angiosperm history, followed by the gamma triplication at the base of the eudicots, and then followed by later independent WGDs within some lineages in which RNS evolved. The ancient WGDs have been named zeta and epsilon in the Amborella Genome Project et al. (2013), and the gamma triplication (also called the γ paleohexaploidy) from early in angiosperm history (The French–Italian Public Consortium for Grapevine Genome Characterization, 2007; Jiao et al., 2012; Ren et al., 2018). The epsilon duplication has been inferred to predate the angiosperm origin, and affecting e.g. *Amborella* and water lilies as well as all eudicots (Zuccolo et al., 2011; Amborella Genome Project et al., 2013). The hypothesized zeta duplication is yet older, predating the gymnosperm origin, with an estimated date of more than 300 Mya (Jiao et al., 2011; Amborella Genome Project et al., 2013), although some aspects of its occurrence and timing remain controversial. The gamma triplication has been observed in most eudicots (The French–Italian Public Consortium for Grapevine Genome Characterization, 2007; Jiao et al., 2011; International Peach Genome Initiative et al., 2013). Zuntini et al. (2024) place the origin of the eudicots (and hence the gamma WGD) near the Late Jurassic and Early Cretaceous Periods, approximately 145 Mya. Magallón et al. (2015) date the eudicot origin to between 133 and 130 Mya.

More recent WGD events (**Figure 1**) are known to have occurred in some lineages with RNS, including within the Fabaceae, prior to the diversification of the Papilionoideae subfamily, and another distinct WGD affecting the Caesalpinioideae, likely as an allopolyploid event that predated the Caesalpinioideae diversification (Stai et al., 2019, Stai et al., 2025; Koenen et al., 2021; Zhao et al., 2021).

Considering these WGD events together, a gene in the Papilionoideae or Caesalpinioideae that retained every WGD-derived duplicate and acquired no additional private duplications would have increased to a copy number of 24: 2x2x3x2 (zeta x epsilon x gamma x papilionoid-or-caesalpinioid), with a further duplication in soybean. In *Arabidopsis*, the successive alpha and beta duplications and the gamma triplication would in principle have expanded the gene set to ~324,000 (27,000×2×2×3), but fractionation has reduced it back to ~27,000 (Panchy et al., 2019). In addition to WGDs, small-scale duplications (SSDs), including tandem duplications (adjacent paralogs) and proximal local duplications (non-adjacent paralogs within 1 Mb on the same chromosome), further contribute to gene family copy number expansion.

Although such an increase in copy number might seem to provide abundant material for neofunctionalization among a family of genes, the timing of the known WGDs presents a puzzle relative to the origin of RNS. If the zeta and epsilon duplications occurred prior to the origin of the angiosperms, and the gamma triplication occurred prior to the diversification of the eudicots—and if this twelve-fold ramification was important in the origin of RNS, then why is there a lag time of at least ~25 million years under a single-origin model (from the gamma triplication to the NFNC ancestor), and perhaps as much as ~65 million years under a multiple-origins model, in which legumes assembled nodulation independently? (Note that here and subsequently, we use the Magallón et al. (2015) estimate for the date of the legume origin rather than the earlier date of ~145 Mya for legume origin from the Angiosperm “young tree” in Zuntini et al. (2024), largely for consistency with the large body of prior literature on the legume origin, [e.g (Lavin et al., 2005; Bruneau et al., 2008; Magallón et al., 2015; Koenen et al., 2021; Zhao et al., 2021)]. At the same time, since the majority of legume species (in the two largest legume subfamilies) can form root nodule symbioses, while only a few other FaFaCuRo lineages can, the whole-genome duplications that occurred early in the evolution of legumes (in particular, near the origin of the Papilionoideae and Caesalpinioideae; Stai et al. (2025) likely played a key role in amplifying nodulation capacity within the family.

This temporal discrepancy between the gamma triplication and the apparent origin of RNS may be partially explained by the “WGD Radiation Lag-Time Model,” which posits that although WGDs provide abundant genetic material instantaneously, there is often a substantial delay before this genetic redundancy translates into novel, complex traits. During this lag period, duplicated genes accumulate mutations and undergo regulatory changes that eventually lead to phenotypic innovation (Schranz et al., 2012). Predisposing innovations may have long predated the evolution of RNS (Soltis et al., 1995; Kates et al., 2024), arising in or even before the ancestor of the NFNC that was either a “precursor” that allowed nodulation to eventually arise millions of years later (in multiple origins models) or enabled the immediate single origin of nodulation, in which case a precursor is not needed. Notably, the temporal discrepancy is substantially reduced under a single-origin model, which eliminates much of the gap between the precursor trait and the emergence of full nodulation (van Velzen et al., 2019).

The main focus in the present study is on gene duplications. The main source for gene duplications in most gene families is from polyploidy (Cannon et al., 2004; Ren et al., 2018; Panchy et al., 2019; Qiao et al., 2019), because polyploidy affects all genes in the organism (at least initially). It should also be noted that private gene duplications (as well as losses) are common in plants, and are the predominant mechanism driving copy number increases in certain types of gene superfamilies such as the p450 enzymes, NBS-LRR disease resistance genes, glutathione transferases, kinases, and Small Ubiquitin-Like Modifier (SUMO) genes (Cannon et al., 2004; Van de Peer et al., 2009; Hammoudi et al., 2016).

To investigate whether the duplication history of RNS-related genes corresponds to conserved versus lineage-specific recruitment into nodulation pathways, we examine 187 genes, across 18 species (19,245 genes in total), organized into 175 gene superfamilies, that have been reported to have important roles in RNS (Roy et al., 2020; Liu et al., 2024; Zhang et al., 2024b). In many cases, loss-of-function phenotypes have been reported for the genes in question (90 genes, per Roy et al., 2020; see **Supplementary Data**). By integrating superfamily phylogenies, duplication histories, and expression patterns, we assess whether ancient WGDs and more recent local duplications contributed differently to gene recruitment and specialization in nodulation.

## Materials and methods

### Taxon sampling choices

Species selected for analysis were chosen based on (1) available literature regarding gene function in nodulation (primarily *Lotus japonicus*, *Medicago truncatula*, *Glycine max*), (2) comparable gene expression data on nodules and other root and shoot tissues (*Lotus japonicus*, *Medicago truncatula*, *Glycine max*, *Phaseolus vulgaris*, *Chamaecrista fasciculata*), (3) non-nodulating comparisons in the legumes (*Senna tomentosa*, *Bauhinia variegata*, *Phanera championii*, *Cercis canadensis*, *Sindora glabra*), (4) representation from the legume subfamilies, given available chromosome-scale genome assemblies (four of the six subfamilies are represented; Dialoideae and Duparqueteae lacked genome assemblies at the time of analysis). Outgroup species were chosen to represent a range of distances from the legumes, from *Quillaja saponaria* [from Quillajaceae - which, along with Surianaceae and Polygalaceae, also in Fabales, are the closest outgroup families to the legumes; (Koenen et al., 2020; Zuntini et al., 2024)]; then three additional non-legume rosid species: *Parasponia andersonii* (Cannabaceae; with rhizobial nodulation) and *Trema orientale* (Cannabaceae; non-nodulating) (Van Velzen et al., 2018) and *Prunus persica* (Rosaceae, non-nodulating). Lastly, to help provide context for interpreting older divergence times and early WGD events, *Arabidopsis thaliana* (Eurosid II/Malvid clade, distinguishing it from the legumes in Eurosid I/Fabid clade), and then *Vitis vinifera* (Eurosid clade, but sister to all other rosids; Wang et al., 2009). We intentionally limited the taxon set to this relatively small collection (18 species) in order to make analysis and visualization more tractable than would have been the case with denser sampling. In particular, the synteny analyses are challenging to visualize with large numbers of species, since they include WGD-derived duplications as well as all of the selected species.

### RNA-sequencing data collection and expression quantification

To investigate tissue-specific expression of genes involved in RNS, RNA-seq datasets were assembled and processed from four legumes: *M. truncatula*, *G. max, P. vulgaris*, and *C. fasciculata.* In each case, the focus was on a curated set of 175 RNS-related superfamilies (Roy et al., 2020; containing 187 genes with reported RNS function). For these superfamilies, relative expression levels were estimated across nodules, roots, and shoots, for the four indicated species. In the case of *G. max* and *P. vulgaris*, only normalized fragments per kilobase per million (FPKM) values were available at the time of analysis; consequently, direct statistical comparison was not applied. Instead, relative expression percentages were computed to enable cross-species comparison of expression trends. Because developmental stages cannot be precisely matched across species, expression values were averaged within each tissue category (nodule, root, and shoot) and used to compare broad tissue preferences rather than fine-scale or time-resolved symbiotic dynamics. All RNA-seq datasets used in this study, including tissue category, original sample identifiers or developmental time points, and their corresponding public accession numbers or source resource identifiers, are provided in **Supplementary Table 3**.

For *M. truncatula* and *C. fasciculata*, raw RNA-seq reads were obtained from de Bang et al. (2017) and the Legume Information System datastore (https://data.legumeinfo.org/Chamaecrista/fasciculata/transcriptomes/), respectively. These reads were quantified using Salmon (v1.10.0) (Patro et al., 2017) in quasi-mapping mode (-l A, --validateMappings). Transcript-level abundances were summarized to gene-level counts using tximport (v1.34.0) (Soneson et al., 2016), and normalization and differential expression analysis were performed using edgeR (v4.4.2) (Robinson et al., 2010) with TMM normalization and generalized linear modeling. The mean expression and standard errors were computed for each group, and the relative expression was calculated by dividing each tissue’s mean value by the gene’s total expression across all tissues. While differential expression was evaluated during the analysis, only relative expression values were utilized for subsequent comparisons. All analyses were conducted in R (v4.4.2).

For *G. max* and *P. vulgaris*, pre-normalized FPKM expression matrices were obtained from public resources (Phytozome v13 release of the Wm82.a4.v1, https://data.jgi.doe.gov/ and Pv Gene Expression Atlas (O’Rourke et al., 2014), respectively). In both cases, samples were grouped by tissue, and mean expression was calculated per gene across replicates. The relative expression of genes in different tissues was derived by calculating the proportion of total gene expression attributable to each tissue.

### Construction and annotation of superfamilies

The gene superfamilies used in this study were constructed by merging legume-focused gene families described in Lee et al. (2024), available at https://data.legumeinfo.org/LEGUMES/Fabaceae/genefamilies/legume.fam3.VLMQ/, into superfamilies when the consensus sequences of the legume.fam3.VLMQ families met the following homology criteria, using mmseqs easy-cluster (Steinegger and Söding, 2017): a protein identity threshold of >= 40% and mutual alignment coverage threshold of >= 50%. The resulting merged sequence files, each consisting of the sequences from one or more of the legume.fam3.VLMQ families, were then each aligned using famsa (Deorowicz et al., 2016), and the alignments were used to calculate a hidden Markov model (HMM), using hmmbuild from the Hmmer package (Finn et al., 2011) for each superfamily. The number of superfamilies at this stage was 14378, down from 25682 families in the legume.fam3.VLMQ set. The 18 gene sets used in this study were then searched against the superfamily HMMs using hmmsearch. The sequences in each superfamily were then realigned to the respective HMM using hmmalign, the alignment was trimmed to HMM match-state characters. Trees were calculated from the trimmed alignments using Fasttree v. 2.1 (Price et al., 2010). The resulting superfamily files (protein multifasta files, HMMs, alignments, trimmed alignments, and phylogenetic trees with bootstrap support values) are available at https://agdatacommons.nal.usda.gov/articles/dataset/Data_from_Impacts_of_gene_duplication_in_the_evolution_of_symbiotic_root_nodule_symbiosis/30142387.

The correspondences between the 187 sequences reported in Roy et al. (2020) as having RNS functions, and the superfamilies identified above, query sequences were collected for each of the Roy et al. (2020) set and used as hmmsearch queries against the superfamily HMMs above, with an E-value threshold of 1e-10. To check superfamily consistency and to determine functional annotations, the superfamilies constructed above were compared against the PANTHER v19 dataset (Mi et al., 2019), using the pantherScore2.2 utility. The resulting functional descriptions are included in **Supplementary Data**.

To evaluate whether the distribution of per-superfamily gene counts differed among species, we performed non-parametric statistical tests in R (v4.4.2). We used a Kruskal–Wallis test to assess overall differences across species and calculated the effect size using epsilon-squared (ϵ²). Then, we applied pairwise Wilcoxon rank-sum tests with Benjamini–Hochberg correction to identify specific interspecific contrasts. The complete matrix of Benjamini–Hochberg (BH) adjusted q-values is provided in **Supplementary Table 2**.

### Determination and categorization of tissue expression bias

The presence of tissue-specific bias was determined by calculating the fold changes in mean relative expression values across the three tissues. A two-fold (≥2) threshold was implemented to ascertain a significant bias toward a specific tissue, a simple and widely used cutoff in comparative RNA-seq analyses that provides a conservative summary of predominant tissue preferences (The MicroArray Quality Control (MAQC) Consortium, 2006; Love et al., 2014). Initially, gene-level biases were determined by direct comparison of relative expression values, and each gene was classified into one of four simplified tissue categories (nodule, root, shoot, or neutral) based on the highest expression level. Subsequently, we calculated superfamily-level biases by averaging tissue expression values for all genes within each superfamily per species. In instances where the highest tissue expression was at least twice that of any other tissue, the superfamily was classified as biased toward that tissue. Conversely, the remaining portion was designated as “neutral”. Because these superfamily-level values are based on mean expression across paralogs, they provide a coarse summary of overall trends and can obscure sub-functionalization among recent duplicates; accordingly, family- and clade-level biases are interpreted as broad trends, whereas gene-level heatmaps and example phylogenies are used to highlight divergent tissue-specific expression among individual paralogs.

We employed a comparative analysis of tissue biases across species to categorize superfamilies into biologically meaningful groups. Superfamilies were classified into five primary categories based on bias conservation or divergence: (1) Those that are conserved across all species; (2) Those that are conserved within the three Papilionoideae species (PAP) but distinct from the caesalpinioid, *Chamaecrista* (CAE); (3) Those that are mixed within PAP and partially overlapping with CAE; and (4) Those that are mixed within PAP and distinct from CAE. (5) Insufficient or missing data or neutral patterns. The data processing, bias calculations, and categorization were performed using custom R scripts (dplyr v1.1.4, tidyr v1.3.1 packages).

### Definition of legume gene clades

For the purpose of summarizing duplication patterns across large gene superfamilies, we defined a legume gene clade as a phylogenetic group in which most sequences were derived from legumes and formed a coherent monophyletic assemblage, even if a small number of non-legume sequences were nested within the clade. Legume clades were identified as collections of (1) legume-family sequences adjacent in the listing of genes in a rooted gene family phylogeny, (2) allowing for a single included non-legume sequence flanked by legume sequences. These assignments were made using the formula in column “legume clade” in worksheet “genes in tree order” in **Supplementary Data S1**.

## Results

### Gene family and superfamily composition and structure

The species tree of the 18 sampled legumes and outgroups is shown in **Figure 1** and provides the phylogenetic framework for the cross-species comparisons that follow. Of the 187 genes we examined that are associated with published RNS functions, these fell into 175 superfamilies (**Table 1**). This reduction indicates that some superfamilies contain multiple RNS-related genes. Of those 175 superfamilies, 70% (122/175) comprised multiple “legume gene clades” (defined in Methods). A quarter of the superfamilies (43/175) contain six or more legume gene clades, consistent with multiple ancient WGDs and/or ancient local duplications, and subsequent retention of most gene copies (**Supplementary Figure 1**).

**Table 1:** Representative legume gene superfamilies associated with RNS. Complete list for all 175 superfamilies is available in **Supplementary Data**.

Legume Superfamily ID	Representative gene symbols	PANTHER superfamily name	No. of species	No. of genes	Legume clade count	Seq. count	Representative medicago gene	Figure no.	Nodule phenotype (Roy et al., 2020)
LegSF.fam3.00582	MtGA2ox10	GIBBERELLIN 2-BETA-DIOXYGENASE 6	18	76	4	14	Medtr4g074130	5	n/a
LegSF.fam3.00681	MtDNF2	F14J22.5 PROTEIN	18	132	6	76	Medtr4g085750	8	Fix-, Fix*
LegSF.fam3.02030	MtBHLH476 & GmbHLHm1	SYMBIOTIC AMMONIUM TRANSPORTER	18	136	4	90	Medtr5g014520	6	Fix+
LegSF.fam3.02933	LjNIN	PROTEIN NLP2	18	56	2	12	Medtr5g099060	4	Fix-
LegSF.fam3.03116	LjNFR5 & NFP	SERINE_THREONINE RECEPTOR-LIKE KINASE NFP	16	49	2	8	Medtr5g019040	9	n/a
LegSF.fam3.14284	LjNFR1 MtLYK3 & LjNFRe	LYSM RECEPTOR KINASE K1B	18	72	1	49	Medtr5g086310	10	Fix-
LegSF.fam3.14698	MtSER6	SERPIN-ZX-LIKE	18	183	3	125	Medtr3g101010	7	Fix+/-

Local duplications are numerous in many of the superfamilies. From a phylogenetic context, it is possible to determine whether such duplications are recent or old relative to speciation and WGD-derived duplications. Of the 175 superfamilies we examined, 148 (84.6%) have at least one local gene duplication (where “local duplicates” were defined as paralogs located within 1 Mb on the same chromosome and were further subdivided into tandem duplicates (immediately adjacent paralogs) (see **Supplementary Data S1**, sheet “SFamily names and counts”) and proximal duplicates (non-adjacent paralogs within this 1-Mb window)), and 34.2% of all genes across all superfamilies (6589 of 19245) have a local paralog (see **Supplementary Data S1**, sheet “SFamily names and counts”). This high proportion is affected by the larger superfamilies, many of which have expanded predominantly through local duplications.

Ancient duplications pre-dating the legume origin (either segmental/WGD or local SSDs, including tandem and proximal duplicates) may be inferred from the presence of multiple clades with similar species membership of legume and non-legume outgroup species (**Figure 1C**). Ancient local duplications are distinguished from segmental duplications by the presence of genomically proximal genes, in several species, found in distinct clades with a distant common ancestral gene (**Figure 1D**). An example of such a conserved ancient local or tandem duplication is found in the DNF2 family (LegSF.fam3.00681), clades 3 and 4 each contain a full suite of legume sequences as well as nonlegume outgroup species; and for most species, the two clades are comprised of sequences with locally derived paralogs. This can be seen both in the corresponding synteny plots and in the gene IDs, which contain positional information. Looking just at the chromosomes, each clade has *Glycine* sequences from both chromosomes 1 and 11 (these reflecting the additional *Glycine* WGD), *Phaseolus* sequences from both chromosomes 3 and 11 (reflecting the papilionoid WGD), *Medicago* sequences from chromosome 4, *Aeschynomene* sequences from chromosome 5, *Prunus* sequences from chromosome 7, etc. From this combined phylogenetic and syntenic information, we infer a local paralogous duplication in the common gene progenitor, predating the eudicot diversification. Among the 175 superfamilies, 24 of these (13.7%) have ancient, retained local gene duplications – identified when at least six species have paralogous copies located within 1Mb of one another on the same chromosome, in different legume clades (clades containing only legume sequences and bounded by non-legume sequences).

The largest of the 175 superfamilies, consisting of cytochrome p450 genes (specifically flavone synthase genes), contains 878 genes and 21 legume clades (**Supplementary Figure 1**). Other large superfamilies include NRT1/PTR peptide transporters (697 genes and 27 legume clades), and Ras-related GTPases (480 genes and 19 legume clades). The largest superfamilies tend to have both numerous retained duplications derived, apparently, from ancient WGDs, as well as from local duplications. For example, in the cases of the cytochrome P450 genes, of 878 genes, at least six legume clades derive from retained ancient local gene duplications that predate the origin of the legumes; and 229 P450 genes in this superfamily occur within 1 MB of at least one other gene in the superfamily, indicating that the high copy number in the superfamily is due both to retention of WGD-derived duplications and expansion of local clusters. The occurrence of many p450 genes in genomic clusters likely facilitates the adaptive evolutionary responses to processes including hormonal and defense responses and secondary metabolite biosynthesis; and in turn helping plants to respond to herbivory, pathogen attack, and environmental stresses (Tang et al., 2024; Huang et al., 2025; Shi et al., 2025). In the context of nodulation, genes in the GmIFS1 & MtFNSII family (LegSF.fam3.00003) convert flavanones into other flavone derivatives, which are essential signaling molecules that induce Sinorhizobium meliloti to initiate nodule formation (Zhang et al., 2007). Another family examined in detail in this paper, the SER6 family (LegSF.fam3.14698), exhibits dramatic expansion via local duplications. Recent duplications are inferred from sequence similarity and location (from proximity in the phylogeny and in genomic position).

### Variation in superfamily sizes and composition

**Figure 2:**
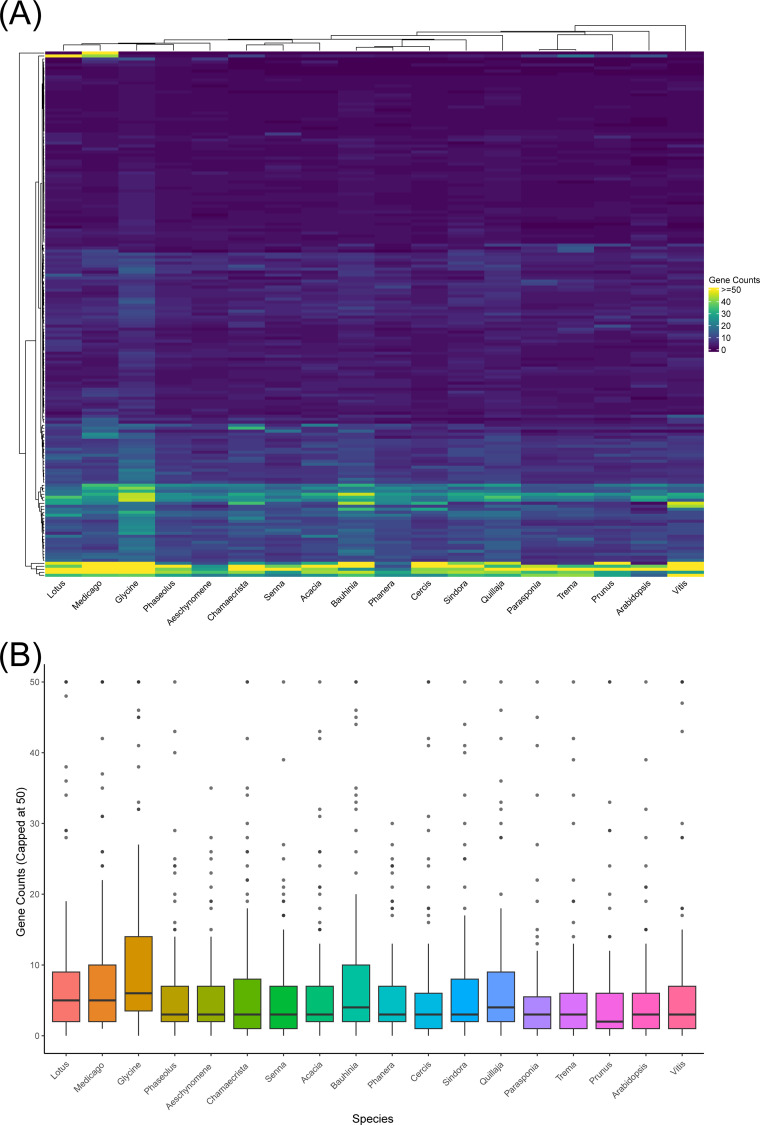
Gene count variation per superfamily across 18 species. **(A)** Heatmap illustrating the variation of gene counts per superfamily (capped at 50). Each row represents a gene superfamily, and each column corresponds to a species. Colors indicate gene count abundance, ranging from dark purple (low counts) to bright yellow (high counts; ≥50). The numerical data used to generate this heatmap are available in **Supplementary Data**. **(B)** Boxplots summarizing the overall gene count distributions for each species (capped at 50). Each boxplot shows median values (horizontal lines), Q1 and Q3 (box edges), and data extremes (whiskers). Points beyond whiskers indicate outliers, representing superfamilies with exceptionally high gene counts.

To help visualize the interspecific variation in gene family sizes associated with RNS, we plotted the number of genes per superfamily across 18 nodulating and non-nodulating legume and outgroup species. In the heatmap (**Figure 2A**), an upper limit of 50 was applied to mitigate distortion from exceptionally large gene families. This approach facilitated the detection of both unexpanded and expanded superfamilies. The heatmap illustrates that a substantial proportion of RNS-related superfamilies are broadly conserved, with comparable gene family sizes across species; but there are notable exceptions. The hierarchical clustering of families on the vertical axis of **Figure 2A** is influenced both by family size (e.g. larger families toward the bottom) and by expansion patterns in some species (e.g. near the top of **Figure 2A**, the nodule-specific cysteine rich (NCR) family (LegSF.fam3.00591) found exclusively in *Medicago*, or the serpin-like SER6 family (LegSF.fam3.14698) found predominantly in *Medicago* and *Lotus*). The horizontal axis reflects the species tree order shown in **Figure 1**.

The boxplots offer a summary of the distribution of gene counts per superfamily across species (**Figure 2B**). The interquartile ranges of most taxa broadly overlap, and the long upper tails reflect the presence of a few very large superfamilies that contribute disproportionately to total counts. A Kruskal–Wallis test revealed statistically significant differences among species (χ² = 129.9, df = 17, p < 0.001; Epsilon² = 0.036). Further analysis using pairwise Wilcoxon tests with BH adjustment showed that *Glycine* differs significantly from several other taxa, including *Lotus, Medicago, Phaseolus, Aeschynomene*, and *Chamaecrista* (all q < 0.01). This finding is consistent with *Glycine*’s recent lineage-specific WGD. Additional significant contrasts were detected, particularly in comparisons involving *Lotus, Medicago*, and *Bauhinia* against multiple non-legume outgroups (e.g., *Lotus–Prunus*, *Medicago–Prunus*, and *Bauhinia–Parasponia*; all q < 0.001). In contrast, most other legumes—such as *Medicago, Phaseolus*, and *Lotus* when compared directly with one another—did not differ significantly, reflecting their shared Papilionoideae duplication. *Cercis* also did not differ significantly despite lacking a lineage-specific WGD, suggesting that the combined effects of retained ancient duplicates and ongoing small-scale duplications may have produced comparable superfamily sizes. Thus, comparable superfamily sizes can arise either through ongoing SSDs in lineages lacking recent WGDs or through differential post-duplication loss following WGD events. The 175 superfamilies analyzed here are all associated with RNS; however, there is no consistent evidence that they are disproportionately expanded in nodulating legumes. In fact, reductions are seen in some taxa, such as *Prunus* and *Trema*. According to a single-origin framework, these taxa represent secondary loss of nodulation. Key nodulation genes, including NIN and, in some cases, RPG, are absent in these taxa (Griesmann et al., 2018; Van Velzen et al., 2018). Overall, the pattern suggests that these superfamilies are not expanded relative to non-nodulating lineages. The complete matrix of pairwise BH-adjusted q-values is provided in **Supplementary Table 2**.

### Gene presence-absence patterns in the RNS pathway

**Figure 3:**
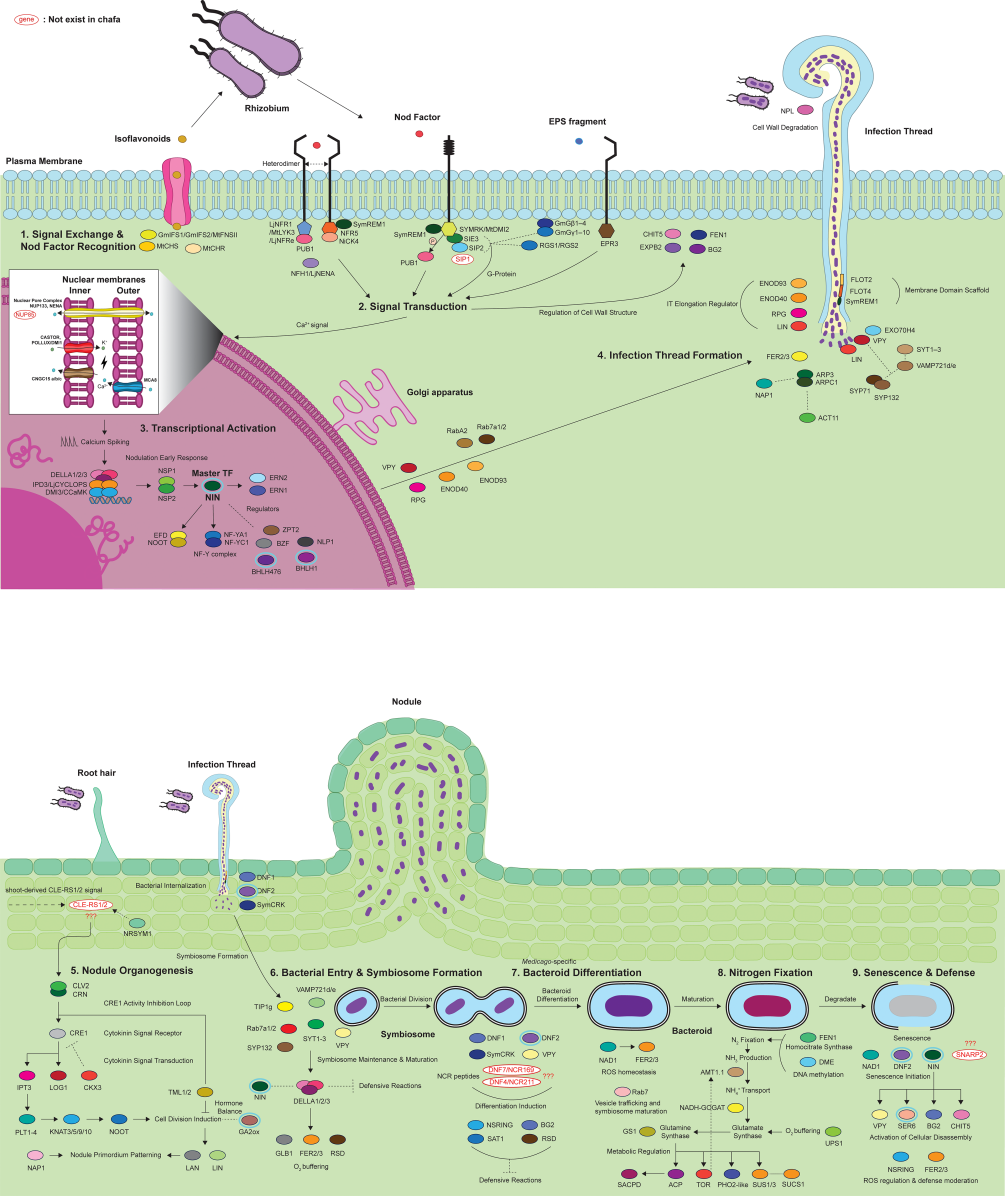
Molecular mechanisms of root nodule symbiosis (RNS). This diagram summarizes the RNS pathway into nine sequential steps. Most of these steps represent broadly conserved processes across legumes, but some features are lineage-specific. For example, bacteroid differentiation (step 7) is characteristic of *Medicago* and related papilionoids: (1) Signal exchange and Nod factor perception, involving isoflavonoid secretion and receptor-mediated recognition (NFR1 & NFR5). (2) Intracellular signaling, characterized by calcium oscillations through CASTOR/POLLUX channels that activate CCaMK-dependent pathways. (3) Activation of transcriptional networks, where nuclear calcium signals trigger key transcription factors such as NSP1, NSP2, NIN, and ERN1. (4) Formation of infection threads, regulated by membrane-domain proteins (e.g. FLOT2, FLOT4), as well as membrane fusion complexes, such as SYP132. (5) Initiation of nodule organogenesis, which is directed by the signaling of cytokines and the control of cell proliferation by CLE peptides. (6) Bacterial entry and symbiosome establishment, which is coordinated via the formation of the symbiosome membrane and associated proteins, such as SYP132. (7) Bacteroid differentiation, which is promoted by NCR peptide signaling and Rab7-dependent vesicle trafficking. (8) Active nitrogen fixation, requiring key enzymes and proteins responsible for homocitrate biosynthesis (FEN1), protein maturation (DNF1), and DNA methylation processes (DME). (9) Nodule senescence and defense responses are mediated by SNARP2 and NAD1 proteins that control the balance of reactive oxygen species (ROS) and overall nodule longevity. Five genes (SIP1, CLE-RS1/2, DNF4/NCR211, DNF7/NCR169, and SNARP2) labeled in red indicate those not identified in *Chamaecrista*. TBLASTN searches (e-value cutoff of 1e-5) produced only fragmentary hits for DNF4/NCR211 (70-82% identity and short alignments) and SIP1 (37.5% identity over 88 amino acids) (**Supplementary Table 1**). Genes used for phylogenetic analysis of RNS (NIN, GA2ox, bHLH transcription factors, SER6, and DNF2) are highlighted with a light blue ring around them.

Four species were prioritized for close examination: *Medicago, Glycine, Phaseolus*, and *Chamaecrista*. These species were chosen due to the availability of transcriptome datasets, which enabled robust inference of gene expression and conservation. To understand how evolutionary changes in RNS-related genes occur, we created a simplified diagram of the typical legume–rhizobium symbiosis process based on arguably the best-studied nodulation symbiosis, that of *Medicago truncatula*. This diagram has nine stages, from the first signs of a signal to the final stage of nodule aging (**Figure 3**). While most of these stages represent conserved modules across legumes, bacteroid differentiation (step 7) is a lineage-specific feature restricted to certain papilionoids including *Medicago*. Importantly, the genes shown in this diagram do not represent the complete set of 187 RNS-associated genes analyzed in this study. Instead, they depict a curated subset of representative genes selected to illustrate the major functional modules of the nodulation pathway. All of the genes shown belong to superfamilies derived from the RNS gene list of Roy et al. (2020), which formed the basis of the 175 superfamilies examined here. Although *Chamaecrista* has been included in broader phylogenomic and phylotranscriptomic surveys (e.g., Zhang et al., 2024b), it has remained underrepresented in pathway-focused, species-resolved comparisons of nodulation genes and expression. Therefore, its phylogenetic position within Caesalpinioideae motivated a closer examination here. The presence/absence patterns and gene copy numbers underlying these comparisons are summarized in **Figure 2A** and provided in full in **Supplementary Data S1** (sheet “gene counts in superfams”), allowing direct visualization of species-level variation across the RNS superfamilies.

Five genes (CLE-RS1/2, SIP1, DNF4/NCR211, DNF7/NCR169, and SNARP2) had no identifiable orthologs in the current *Chamaecrista* assembly and are marked in red in the diagram. TBLASTN searches with an e-value of 1e-5 produced only partial matches. DNF4/NCR211 showed short alignments with 70–82% identity across 20–40 amino acids; SIP1 showed a low-similarity match with 37.5% identity across 88 amino acids. CLE-RS1/2, DNF7/NCR169, and SNARP2 produced no significant hits (**Supplementary Table 1**).

These genes span various functional stages, including infection thread formation (SIP1), signal autoregulation (CLE-RS1/2), bacteroid differentiation (DNF4 and DNF7), and nodule senescence (SNARP2). Several of these genes (e.g., DNF4, DNF7, SNARP2) are already known to be restricted to *Medicago* (Roy et al., 2020). Accordingly, their absence in *Chamaecrista* is best interpreted as reflecting lineage-specific innovations in *Medicago*, rather than pathway divergence in the Caesalpinioideae. Since these genes are also absent or not implicated in nodulation in other papilionoid legumes, such as *Glycine* and *Lotus*, a single gain in *Medicago* is a more parsimonious explanation than multiple independent losses. These observations also highlight the highly variable molecular basis of nodulation within the papilionoid lineage, paralleling the well-known diversity in nodule morphology, anatomy, and biochemistry.

Two of the missing genes are CLE-RS1/2 (LegSF.fam3.08718) and SIP1 (LegSF.fam3.12488) (**Table 2**). CLE-RS1/2 homologs are broadly distributed across legume subfamilies. Within Caesalpinioideae, they were detected in *Acacia* but absent in both *Chamaecrista* and *Senna*, indicating variation in gene presence across species within Caesalpinioideae. Conversely, SIP1 is consistently absent from all examined Caesalpinioideae species, including *Chamaecrista*, *Senna*, and *Acacia*. SIP1 homologs are found in other plant groups, such as *Arabis*, *Prunus*, and *Vitis*. They are also found in the Rosales and Cercidoideae lineages. The absence of these genes in *Chamaecrista* is consistent with the possibility that nodulation in this lineage may involve molecular mechanisms that differ from those characterized in papilionoid legumes. This interpretation aligns with models that propose nodulation evolved independently in different lineages. In this case, similarities among nodulation pathways reflect convergence rather than divergence from a shared canonical pathway.

**Table 2:** Subfamily-specific conservation patterns of CLE-RS1/2 and SIP1 gene families.

Subfamily (abbr.)	Species (abbr.)	CLE-RS1/2	SIP1
Papilionoideae (PAP)	*Glycine max* (glyma)	4	2
	*Medicago truncatula* (medtr)	3	1
	*Phaseolus vulgaris* (phavu)	2	2
	*Lotus japonicus* (lotja)	2	1
	*Aeschynomene evenia* (aesev)	1	1
Caesalpinioideae (CAE)	*Chamaecrista fasciculata* (chafa)	0	0
	*Senna tora* (sento)	0	0
	*Acacia crassicarpa* (acacr)	1	0
Detarioideae (DET)	*Sindora glabra* (singl)	2	0
Cercidoideae (CER)	*Cercis canadensis* (cerca)	2	1
	*Bauhinia variegata* (bauva)	0	2
	*Phanera championii* (phach)	0	1
Rosales (ROS)	*Quillaja saponaria* (quisa)	1	1
	*Trema orientale* (treor)	3	1
	*Parasponia andersonii* (paran)	3	1
Outgroups (OUT)	*Arabidopsis thaliana* (arath)	2	2
	*Prunus persica* (prupe)	0	1
	*Vitis vinifera* (vitvi)	0	1

The remaining three genes are DNF4/NCR211, DNF7/NCR16, and SNARP. The DNF4/NCR211 gene is essential for maintaining bacteroid viability after differentiation in *Medicago* (Kim et al., 2015). The DNF7/NCR169 gene is critical for terminal differentiation and nitrogen fixation (Van de Velde et al., 2010; Horváth et al., 2015). A review of existing literature (Van de Velde et al., 2010; Horváth et al., 2015; Kim et al., 2015; Roy et al., 2020) shows that both genes appear to be conserved among Papilionoideae species. However, there are no orthologs or functional studies documented in non-papilionoid legumes, including *Chamaecrista*. Similarly, although SNARP2 is implicated in nodule senescence and reactive oxygen species (ROS) regulation, it remains poorly characterized outside of *Medicago*. The underlying presence-absence data for 18 species are available in **Supplementary Data S1** (sheet “gene counts in superfams”).

### Identification and categorization of tissue-specific expression biases

For simplicity, we use the term “tissue-specific” throughout this section to encompass expression differences between the nodule organ and the root and shoot tissues, while recognizing that the nodule is a distinct organ formed *de novo*. We systematically analyzed and characterized tissue-specific gene expression biases of superfamilies in four legume species (*Medicago*, *Glycine*, *Phaseolus*, and *Chamaecrista*). The PCA was performed once across all four species using centered and scaled relative expression values (prcomp with scale = TRUE in R), ensuring comparable contribution of each tissue variable. A Principal Component Analysis (PCA) based on relative expression data was conducted to define these biases and categorize gene superfamilies by comparing relative fold change between tissues (**Supplementary Figure 2**). PC1 and PC2 explained 41.9% and 19.9% of the total variance, respectively (61.8% cumulative). Examination of the dominant loadings indicated that PC1 primarily captures a contrast between nodule- and shoot-biased expression across species, whereas PC2 reflects a root-versus-nodule contrast. Superfamilies exhibiting strong tissue-specific biases tend to fall toward the periphery of the PCA space, whereas those with weaker or more balanced expression cluster closer to the origin within the two-dimensional representation (PC1 = 0, PC2 = 0).

The distribution of tissue-specific biases differed notably among species. The most prevalent form of expression, nodule-biased expression, was observed in *Medicago* (130), followed by *Phaseolus* (66), *Glycine* (46), and *Chamaecrista* (42). The highest levels of root-biased expression were observed in *Phaseolus* (34), followed by *Glycine* (33), *Chamaecrista* (10), and *Medicago* (7). Shoot-biased expression was most prevalent in *Chamaecrista* (70), followed by *Glycine* (51), *Phaseolus* (11), and *Medicago* (9). The neutral pattern, indicative of comparable expression across multiple tissues, was most prevalent in *Phaseolus* (62), followed by *Chamaecrista* (47), *Glycine* (44), and *Medicago* (29). The higher prevalence of neutral expression indicates that many genes exhibit similar expression levels across various tissues, implying weaker tissue-specific specialization.

A predominance of nodule-biased expression might have been anticipated, since these superfamilies were originally defined in Roy et al. (2020) because they were identified as genes functionally involved, and in many cases essential, in nodulation. However, this pattern was not consistently observed across species. Instead, the data are consistent with the long-hypothesized recruitment of genes and regulatory modules from pre-existing functions. Such recruitment need not be confined to the origin of nodulation, but is also compatible with later refinement of an already established symbiosis (Young et al., 2011; Li et al., 2013). Taken together, this suggests that neofunctionalized paralogues may have been repeatedly incorporated into the symbiotic pathway over millions of years, emphasizing the continuing and dynamic nature of RNS evolution.

We also categorized gene superfamilies based on their expression patterns (**Supplementary Figure 3**).

**Figure 4:**
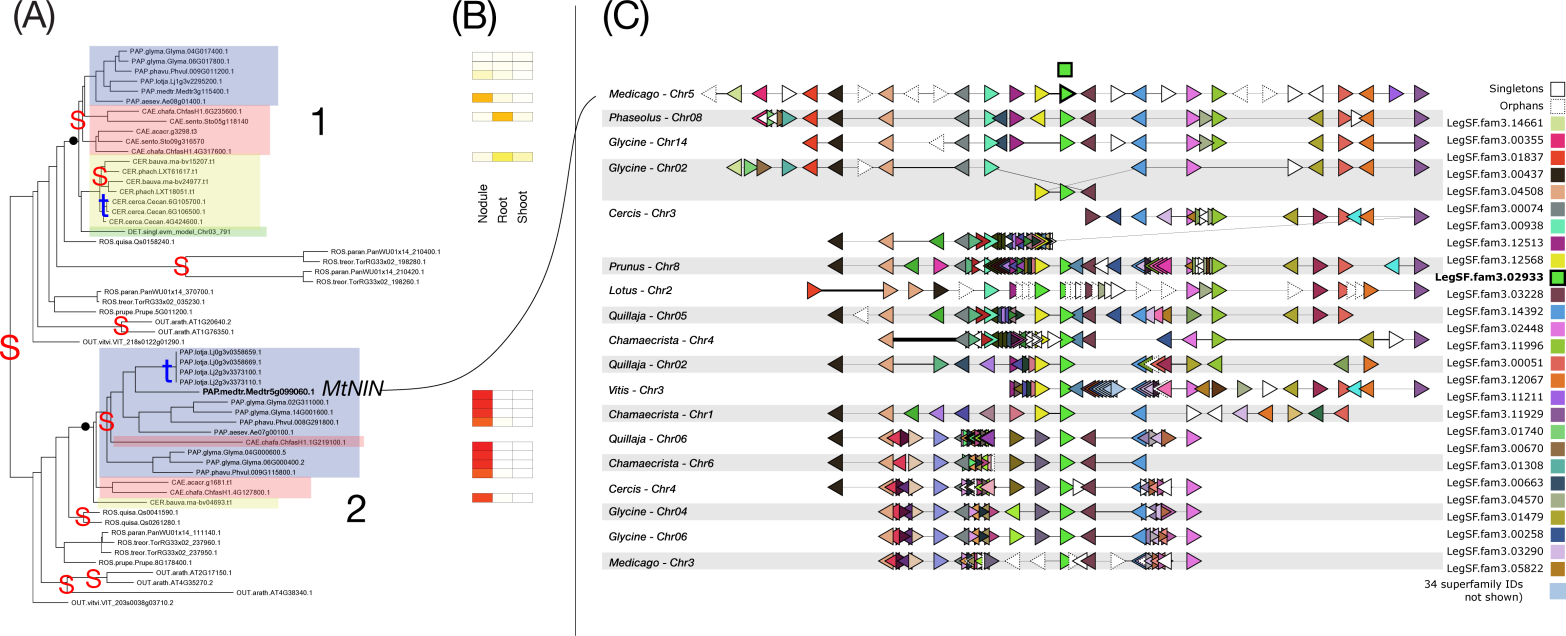
Phylogeny, expression, and synteny for the superfamily of NIN, LegSF.fam3.02933. **(A)** Gene phylogeny. For bootstrap values, see **Supplementary Figure 4**. Legume family clades are indicated with numbers 1-2. Subfamilies are indicated with colors: Papilionoideae (blue), Caesalpinioideae (red), Detarioideae (green), Cercidoideae (yellow). Species not colored are individually labeled with ROS (non-legume Rosid clade; *Quillaja*, *Parasponia*, *Trema*) or OUT (*Arabidopsis* and *Vitis*). Nodes labeled “S” and “t” in the phylogeny indicate, respectively, Segmental (generally WGD-derived) duplications or tandem/local duplications. These are inferred from instances of two clades of legume genes with non-adjacent locations (S) or adjacent locations (t). **(B)** tissue expression for selected species; red indicating high expression, yellow indicating low expression, white indicating no expression. **(C)** Synteny plot around NIN orthologs, for selected genera (*Glycine*, *Medicago*, *Lotus*, *Phaseolus*, *Cercis*, *Chamaecrista*, *Quillaja*, *Prunus*, *Vitis*). Each row represents the genes (triangles) from a genomic region from the indicated species and chromosome. The query for this plot was *Medicago* gene Medtr5g099060 from clade 2 in the NIN phylogeny. The remaining tracks were identified and aligned based on similar gene content. The NIN homologs are indicated in green, in the alignment columns marked at the top with a green box.

**Figure 5:**
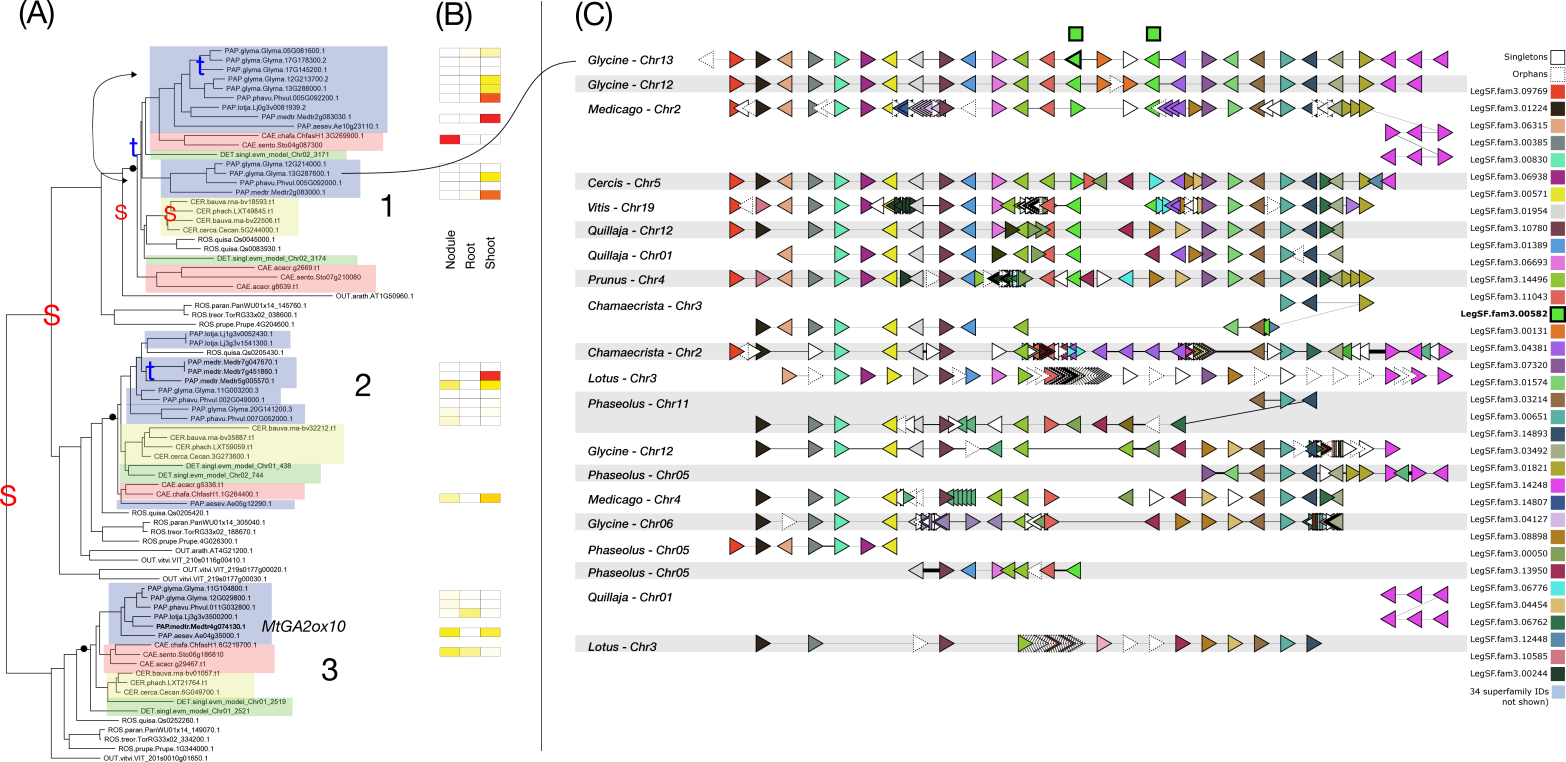
Phylogeny, expression, and synteny for the superfamily of GA2ox, LegSF.fam3.00582. **(A)** Gene phylogeny. For bootstrap values, see **Supplementary Figure 5**. Legume family clades are indicated with numbers 1-3. Subfamilies are indicated with colors: Papilionoideae (blue), Caesalpinioideae (red), Detarioideae (green), Cercidoideae (yellow). Species not colored are individually labeled with ROS (non-legume Rosid clade; *Quillaja*, *Parasponia*, *Trema*) or OUT (*Arabidopsis* and *Vitis*). Nodes labeled “S” and “t” in the phylogeny indicate, respectively, Segmental (generally WGD-derived) duplications or tandem/local duplications. These are inferred from instances of two clades of legume genes with non-adjacent locations (S) or adjacent locations (t). Double-headed arrows indicate inferred tandem duplication events, which link adjacent gene copies within a phylogeny. **(B)** tissue expression for selected species; red indicating high expression, yellow indicating low expression, white indicating no expression. **(C)** Synteny plot around GA2ox orthologs, for selected genera (*Glycine*, *Medicago*, *Lotus*, *Phaseolus*, *Cercis*, *Chamaecrista*, *Quillaja*, *Prunus*, *Vitis*). Each row represents the genes (triangles) from a genomic region from the indicated species and chromosome. The query for this plot was soybean gene Glyma.13G287600 from clade 1 in the GA2ox phylogeny. The remaining tracks were identified and aligned based on similar gene content. The GA2ox homologs are indicated in green, in the alignment columns marked at the top with a green box.

**Figure 6:**
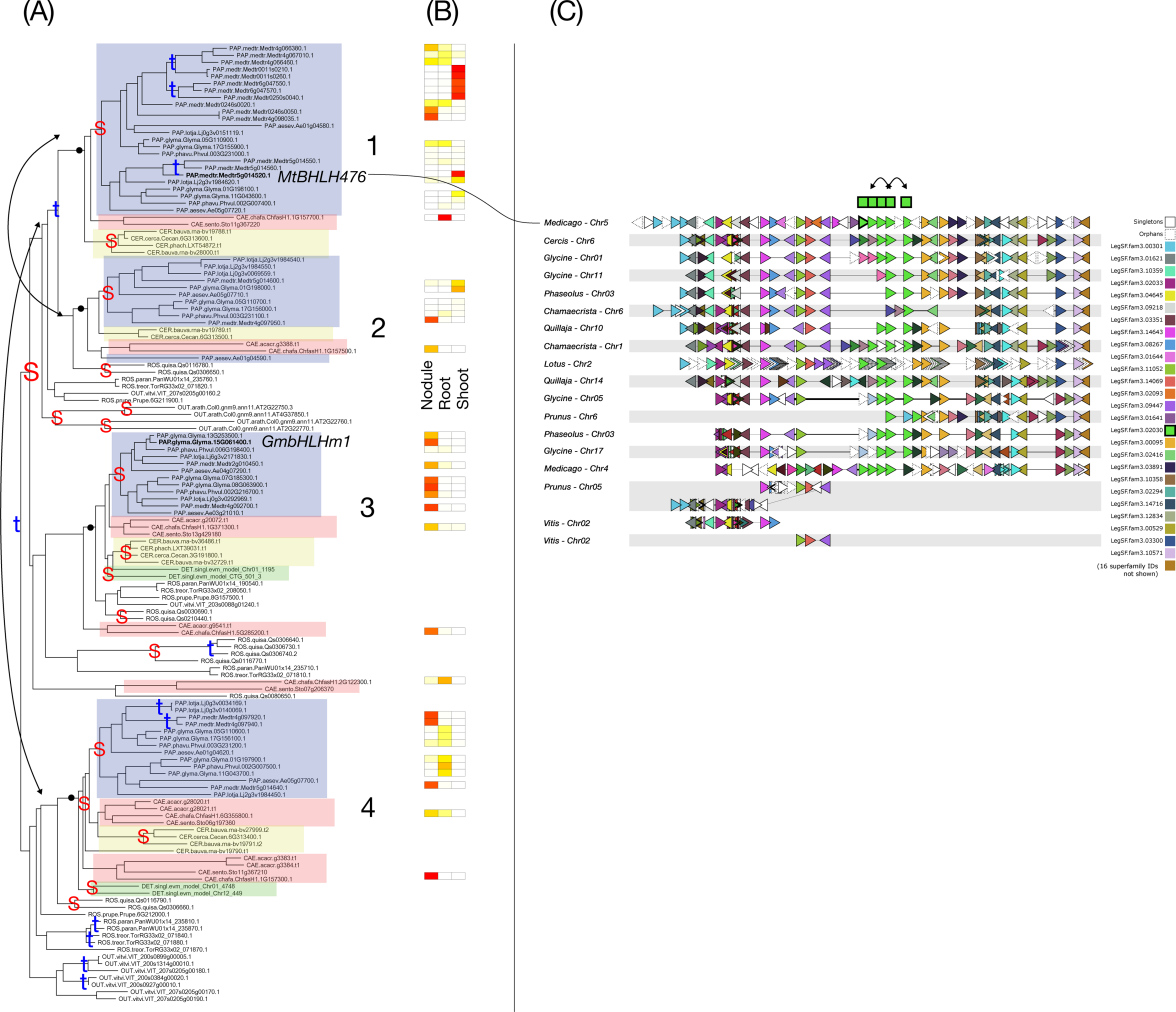
Phylogeny, expression, and synteny for the superfamily of bHLH transcription factors, LegSF.fam3.02030. **(A)** Gene phylogeny. For bootstrap values, see **Supplementary Figure 6**. Legume family clades are indicated with numbers 1-4. Subfamilies are indicated with colors: Papilionoideae (blue), Caesalpinioideae (red), Detarioideae (green), Cercidoideae (yellow). Species not colored are individually labeled with ROS (non-legume Rosid clade; *Quillaja*, *Parasponia*, *Trema*) or OUT (*Arabidopsis* and *Vitis*). Nodes labeled “S” and “t” in the phylogeny indicate, respectively, Segmental (generally WGD-derived) duplications or tandem/local duplications. These are inferred from instances of two clades of legume genes with non-adjacent locations (S) or adjacent locations (t). Double-headed arrows indicate inferred tandem duplication events, which link adjacent gene copies within a phylogeny. **(B)** tissue expression for selected species; red indicating high expression, yellow indicating low expression, white indicating no expression. **(C)** Synteny plot around bHLH transcription factors orthologs, for selected genera (*Glycine*, *Medicago*, *Lotus*, *Phaseolus*, *Cercis*, *Chamaecrista*, *Quillaja*, *Prunus*, *Vitis*). Each row represents the genes (triangles) from a genomic region from the indicated species and chromosome. The query for this plot was *Medicago* gene Medtr5g014520 from clade 1 in the bHLH transcription factors phylogeny. The remaining tracks were identified and aligned based on similar gene content. The bHLH transcription factors homologs are indicated in green, in the alignment columns marked at the top with a green box.

**Figure 7:**
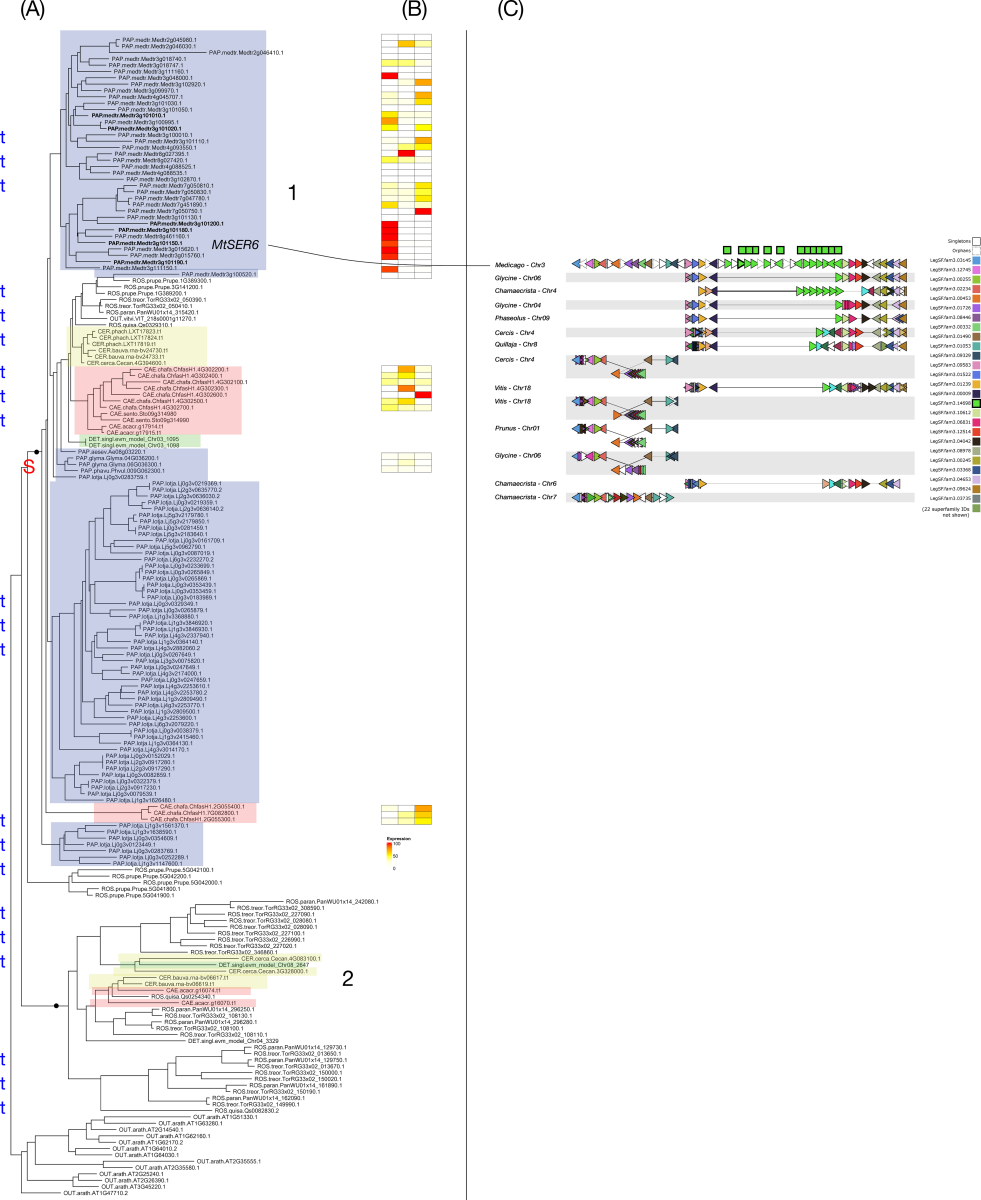
Phylogeny, expression, and synteny for the superfamily of SER6, LegSF.fam3.14698. **(A)** Gene phylogeny. For bootstrap values, see **Supplementary Figure 7**. Legume family clades are indicated with numbers 1-2. Subfamilies are indicated with colors: Papilionoideae (blue), Caesalpinioideae (red), Detarioideae (green), Cercidoideae (yellow). Species not colored are individually labeled with ROS (non-legume Rosid clade; *Quillaja*, *Parasponia*, *Trema*) or OUT (*Arabidopsis* and *Vitis*). Nodes labeled “S” and “t” in the phylogeny indicate, respectively, Segmental (generally WGD-derived) duplications or tandem/local duplications. These are inferred from instances of two clades of legume genes with non-adjacent locations (S) or adjacent locations (t). In this superfamily, tandem duplications have been so frequent that the “t” symbols only indicate multiple tandem duplications are evident in this portion of the phylogeny **(B)** tissue expression for selected species; red indicating high expression, yellow indicating low expression, white indicating no expression. **(C)** Synteny plot around SER6 orthologs, for selected genera (*Glycine*, *Medicago*, *Lotus*, *Phaseolus*, *Cercis*, *Chamaecrista*, *Quillaja*, *Prunus*, *Vitis*). Each row represents the genes (triangles) from a genomic region from the indicated species and chromosome. The query for this plot was Medicago gene Medtr3g101010 from clade 1 in the SER6 phylogeny. The remaining tracks were identified and aligned based on similar gene content. The SER6 homologs are indicated in green, in the alignment columns marked at the top with a green box.

**Figure 8:**
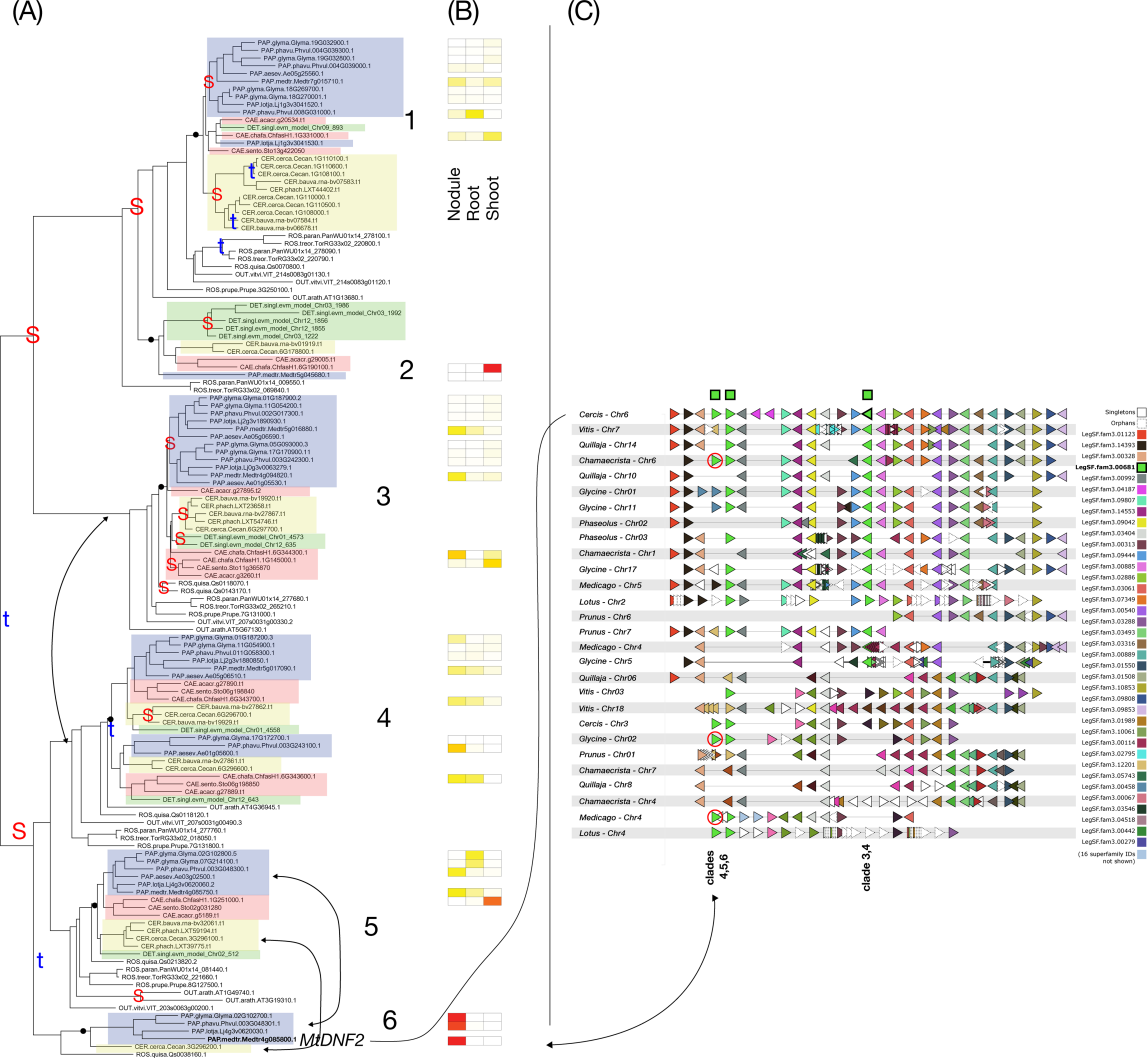
Phylogeny, expression, and synteny for the superfamily of DNF2, LegSF.fam3.00681. **(A)** Gene phylogeny. For bootstrap values, see **Supplementary Figure 8**. Legume family clades are indicated with numbers 1-6. Subfamilies are indicated with colors: Papilionoideae (blue), Caesalpinioideae (red), Detarioideae (green), Cercidoideae (yellow). Species not colored are individually labeled with ROS (non-legume Rosid clade; *Quillaja*, *Parasponia*, *Trema*) or OUT (*Arabidopsis* and *Vitis*). Nodes labeled “S” and “t” in the phylogeny indicate, respectively, Segmental (generally WGD-derived) duplications or tandem/local duplications. These are inferred from instances of two clades of legume genes with non-adjacent locations (S) or adjacent locations (t). Double-headed arrows indicate inferred tandem duplication events, which link adjacent gene copies within a phylogeny. **(B)** tissue expression for selected species; red indicating high expression, yellow indicating low expression, white indicating no expression. The strongest nodule expression is present in the papilionoid genes in Clade 6. Note that *Chamaecrista* is absent from this clade. The *Chamaecrista* genes with strongest nodule expression are in Clade 4. **(C)** Synteny plot around GCV2 for selected genera (*Glycine*, *Medicago*, *Lotus*, *Phaseolus*, *Cercis*, *Chamaecrista*, *Quillaja*, *Prunus*, *Vitis*). Each row represents the genes (triangles) from a genomic region from the indicated species and chromosome. The query for this plot was *Cercis* gene Cecan.3G296200 from clade 5 in the DNF2 phylogeny. The remaining tracks were identified and aligned based on similar gene content. The DNF2 homologs are indicated in green, in the alignment columns marked at the top with a green box. Red circles around the DNF2 homologs on the left side are those with the strongest nodule expression: two papilionoid genes from Clade 6, and two *Chamaecrista* genes from Clade 4.

1. Category 1 includes superfamilies with conserved expression biases across all examined species. As illustrated in **Figure 4** (NIN, LegSF.fam3.02933), there is clear nodule-biased expression across all four species, with some expression noted in roots. These superfamilies likely represent essential, conserved functions related to legume nodulation.
2. Category 2 consists of superfamilies that display conserved expression biases within PAP species (*Medicago*, *Glycine*, and *Phaseolus*), but distinct biases in CAE (*Chamaecrista*). **Figure 5** (MtGA2ox10, LegSF.fam3.00582) serves as a prime example of this category. PAP species exhibit a shoot bias, while CAE (*Chamaecrista*) demonstrates a nodule bias, suggesting the presence of divergent evolutionary pathways between these groups.
3. Category 3 represents superfamilies with mixed expression patterns within PAP species and partial overlap with CAE biases. For instance, **Figure 6** (bHLH transcription factors, LegSF.fam3.02030) demonstrates substantial clade-specific variation, including lineage-specific expression of different duplicated homoeologues in nodulation. In Clade 1, which bears signatures of papilionoid and caesalpinioid whole-genome duplications, *Medicago* and *Phaseolus* express different homoeologous copies in nodules, whereas neither of the corresponding *Glycine* paralogues shows nodule-biased expression. This finding indicates that PAP species have variable expression patterns, suggesting that even closely related legumes can use different regulatory solutions for nodulation. In some cases, these divergent expression profiles resemble those observed in *Chamaecrista*, which may reflect either retention of ancestral expression states under a single-origin model or independent evolutionary changes under a multiple-origins model.
4. Category 4 comprises superfamilies that show mixed biases within PAP species that differ distinctly from those of CAE. **Figure 7** (SER6, LegSF.fam3.14698) shows nodule-biased expression in multiple *Medicago* copies, whereas *Glycine* and *Phaseolus* display more heterogeneous patterns without a consistent tissue bias, and a bias toward shoots in *Chamaecrista*, in contrast to the clear shoot bias observed in *Chamaecrista*. These differences illustrate substantial species-level heterogeneity in expression within this superfamily.
5. Category 5 includes superfamilies with insufficient or neutral expression data – or other complex patterns not meeting the categories above. As shown in **Figure 8** (DNF2; LegSF.fam3.00681), these families often contain multiple retained duplicates with varied expression profiles across lineages. This leads to weak or ambiguous tissue-specific biases. This variability results in an overall neutral classification, despite the known nodulation functions of some members.

**Figure 9:**
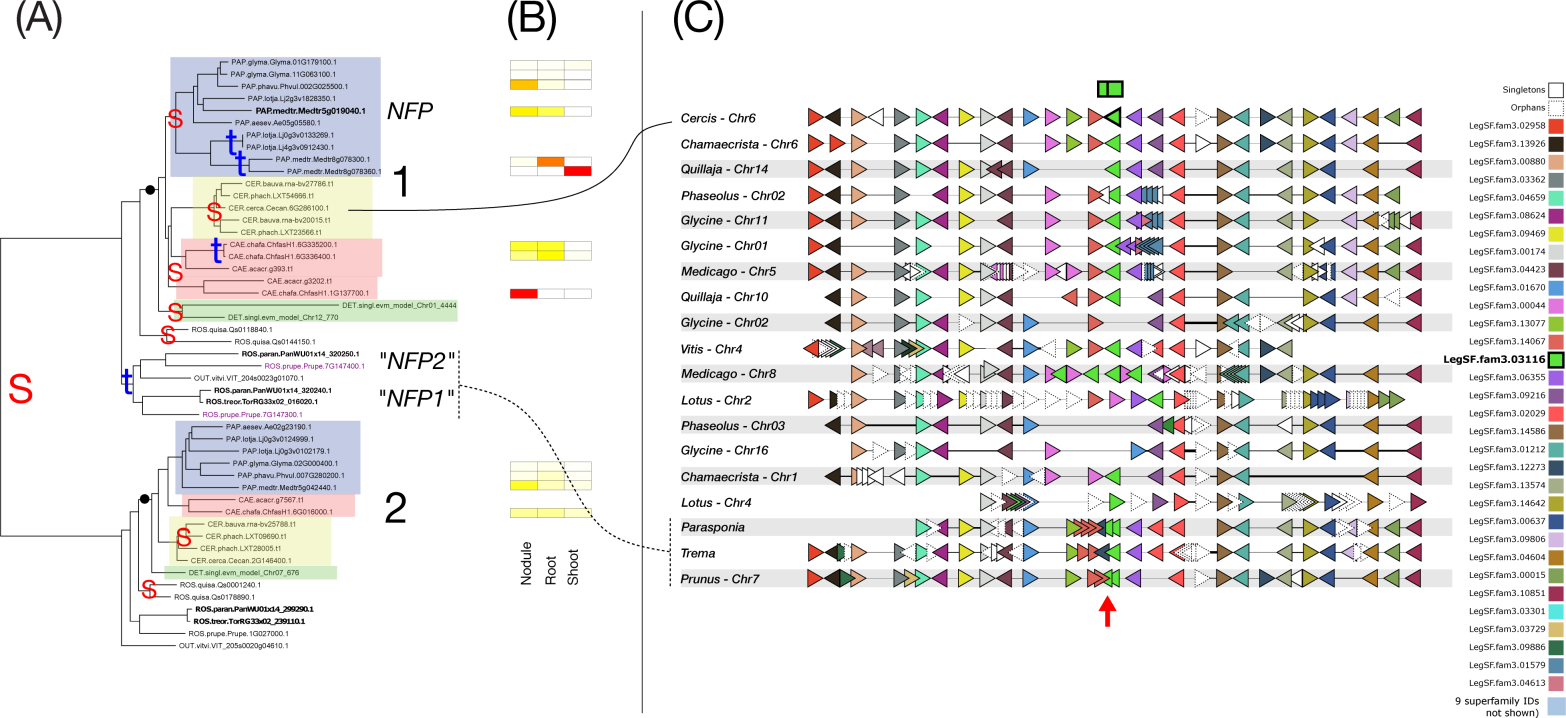
Phylogeny, expression, and synteny for the superfamily of NFP, LegSF.fam3.03116. **(A)** Gene phylogeny. For bootstrap values, see **Supplementary Figure 9**. Legume family clades are indicated with numbers 1-2. Subfamilies are indicated with colors: Papilionoideae (blue), Caesalpinioideae (red), Detarioideae (green), Cercidoideae (yellow). Species not colored are individually labeled with ROS (non-legume Rosid clade; *Quillaja*, *Parasponia*, *Trema*) or OUT (*Arabidopsis* and *Vitis*). Nodes labeled “S” and “t” in the phylogeny indicate, respectively, Segmental (generally WGD-derived) duplications or tandem/local duplications. These are inferred from instances of two clades of legume genes with non-adjacent locations (S) or adjacent locations (t). Note a tandem duplication affecting *Prunus* and *Parasponia*, marked with “*NFP2*” and “*NFP1*” per Van Velzen et al. (2018) and the originally identified *Medicago* gene as “NFP” per Arrighi et al., 2006. The line from that clade leads to the respective regions in *Prunus* and *Parasponia*, where the local duplications are evident (highlighted with a red arrow). **(B)** tissue expression for selected species; red indicating high expression, yellow indicating low expression, white indicating no expression. **(C)** Synteny plot around GCV2 for selected genera (*Glycine*, *Medicago*, *Lotus*, *Phaseolus*, *Cercis*, *Chamaecrista*, *Quillaja*, *Prunus*, *Vitis*, *Prunus*, *Parasponia, Trema*). Each row represents the genes (triangles) from a genomic region from the indicated species and chromosome. The query for this plot was *Cercis* gene Cecan.6G286100 from clade 1 in the NFP phylogeny. The remaining tracks were identified and aligned based on similar gene content. The NFP homologs are indicated in green, in the alignment columns marked at the top with a green box.

**Figure 10:**
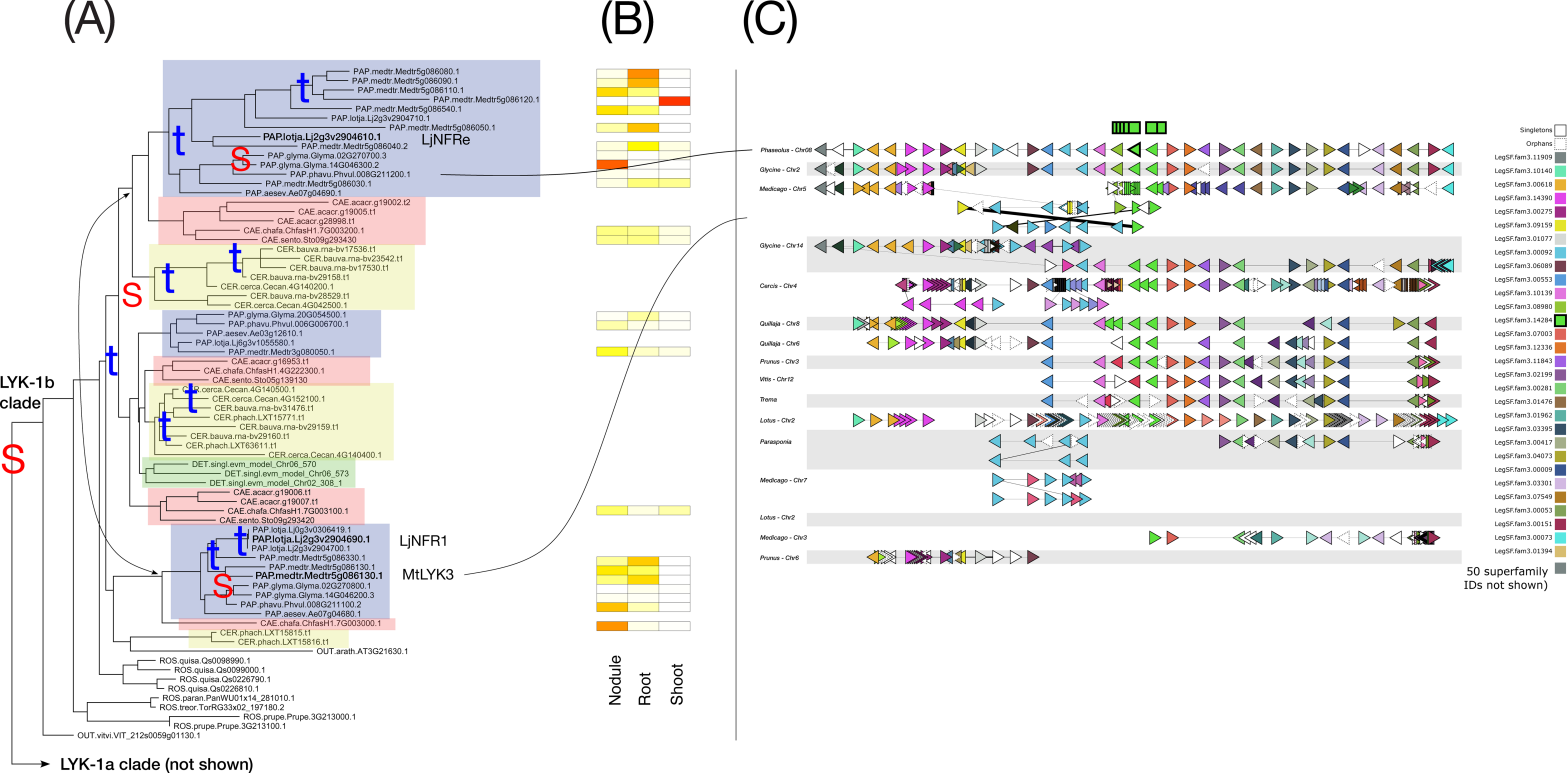
Phylogeny, expression, and synteny for the superfamily of the LYK-1/LjNFR1 LysM receptor kinase family, LegSF.fam3.14284. **(A)** Gene phylogeny. For bootstrap values, see **Supplementary Figure 8**. Subfamilies are indicated with colors: Papilionoideae (blue), Caesalpinioideae (red), Detarioideae (green), Cercidoideae (yellow). Species not colored are individually labeled with ROS (non-legume Rosid clade; *Quillaja*, *Parasponia*, *Trema*) or OUT (*Arabidopsis* and *Vitis*). Nodes labeled “S” and “t” in the phylogeny indicate, respectively, Segmental (generally WGD-derived) duplications or tandem/local duplications. These are inferred from instances of two clades of legume genes with non-adjacent locations (S) or adjacent locations (t). **(B)** tissue expression for selected species; red indicating high expression, yellow indicating low expression, white indicating no expression. **(C)** Synteny plot around GCV2 for selected genera (*Glycine*, *Medicago*, *Lotus*, *Phaseolus*, *Cercis*, *Chamaecrista*, *Quillaja*, *Prunus*, Vitis, *Prunus*, *Parasponia*, *Trema*). Each row represents the genes (triangles) from a genomic region from the indicated species and chromosome. The query for this plot was Phaseolus gene Phvul.008G211200. The remaining tracks were identified and aligned based on similar gene content. The LYK homologs are indicated in green, in the alignment columns marked at the top with a green box.Discussion.

### Phylogeny–expression-synteny integration for selected RNS superfamilies

We examined how duplication modality relates to specialization in seven RNS superfamilies (NIN-LegSF.fam3.02933, GA2ox-LegSF.fam3.00582, bHLH transcription factors-LegSF.fam3.00230, SER6-LegSF.fam3.14698, DNF2-LegSF.fam3.00681, NFP, LegSF.fam3.03116, LYK-1/LjNFR1, LegSF.fam3.14284)) by combining family-level phylogenies with tissue-level expression and gene organization in syntenic regions across species. **Figures 4**–**10** illustrate categories of expression bias (previous section), but also different patterns of gene duplication and retention. All seven figures have the following aspects in common. The phylogeny is on the left panel (A), with “S” and “t” indicating Segmental and tandem duplications, respectively. Segmental duplicates are generally due to WGDs and tandem indicate local duplications. Legume clades are numbered, and legume subfamily groupings are highlighted with colored boxes. The expression patterns, evaluated for *Medicago*, *Glycine*, *Phaseolus*, and *Chamaecrista*, are shown in the central panel (B). The genic environment and synteny plots are given in the right panel (C). In these plots, genes from a superfamily are given a unique color, and aligned to indicate synteny across chromosomal regions from selected species. To keep the synteny plots simpler to interpret, only 9 of the 18 species are included (as indicated in the legends). The superfamily color assignments generally don’t correspond between figures, though the superfamily from the phylogeny is always shown in bright green, with the gene used for the query being shown in the top line, with black border. Locations of the columns with these genes are indicated with a black-bordered green box at the top.

In the NIN (Nodule Inception) family (**Figure 4**), which remains low-copy, a strong, conserved nodule bias is exhibited across the four species for which we evaluated expression. Most strikingly, strong nodule expression is present across all tested nodulating species in legume clade 2, and not in clade 1. Those two clades appear to derive from one of the ancient WGDs – the gamma triplication being the most proximal candidate. However, the critical point is not the precise timing of this duplication, estimated at 115–130 Mya (Vekemans et al., 2012; Gao et al., 2018), but rather that the triplication predates the NFNC ancestor and produced three paralogs present in many eudicots, including lineages in which nodulation never evolved. Therefore, the gamma triplication itself is unlikely to be a key predisposition for nodulation. Rather, subsequent lineage-specific changes, such as the evolution of NIN function in NFNC lineages (Liu and Bisseling, 2020) and the loss of the nitrogen-sensing motif in legumes (Zhao et al., 2021; Zhang et al., 2024b), may have constituted critical enabling steps, at least for legumes. Segmental duplications also appear at the base of the Papilionoideae, consistent with the known WGD predating the radiation of that subfamily (Koenen et al., 2020; Stai et al., 2025), and several tandem duplications are scattered near terminal branches. The tip-proximal tandem events (in *Lotus* and *Chamaecrista*) indicate limited lineage-specific exploration.

Changes in the NIN lineage following the old duplication, indicate neo- or subfunctionalization into specialized roles in the nodule for all members of this clade. These changes include (1) acquisition of a specific protein motif (the FR motif) that stabilizes its dimer interface, allowing it to control nodule-specific symbiotic genes (Nosaki et al., 2026), (2) loss of the nitrate-binding site present in other NIN-like proteins (NLPs) (Gao et al., 2023; Shen and Feng, 2024), and (3) acquisition of the PACE cis-element in the NIN promoter (Cathebras et al., 2026).

Across the GA2ox (Gibberellin 2-oxidase) superfamily (**Figure 5**), genes in the Papilionoideae subfamily are predominantly shoot-biased in legume clade 1, while only limited nodule expression is observed in papilionoid genes in legume clades 2 and 3. In contrast, strong nodule expression in *Chamaecrista* is seen in legume clade 1. The three legume clades are likely derived from an ancient WGD (likely the gamma triplication). The utilization of genes from different WGD-derived clades in the nodule in the Caesalpinoideae (at least in *Chamaecrista*) highlights that strong nodule-biased expression is restricted to a single paralogue in *Chamaecrista*. Alternatively, the observed pattern could be explained by expression shifts following regulatory rewiring, whereby paralogous copies are recruited after nodulation is established. Both possibilities have been discussed previously (Doyle, 1994), and analogous cases are known from other systems. For example, different paralogs are used in grasses sharing the same origin of C_4_ photosynthesis (Mendieta et al., 2024). Also noteworthy in this superfamily is a local duplication that affected genes from *Glycine*, *Phaseolus*, *Lotus*, and *Medicago*. This is evident both in the phylogeny (legume clade 1, two blue boxes) and in the synteny plot (two columns of green-labeled genes from this superfamily, with two duplicated genes on *Glycine* chromosome 13, two on *Glycine* chromosome 12, and two on *Medicago* chromosome 2). *Medicago* Clade 2 genes also illustrate variability in the expression fates of recent paralogues, ranging from strong shoot up-regulation to up-regulation in shoot and nodule to no expression in any of the three organs. Clade 3 includes genes up-regulated in nodules of both *Chamaecrista* and *Medicago*.

The genes in Clade 3 of the GA2ox superfamily, with RNS activity, show clear evidence of neo- or subfunctionalization. While GA2ox genes generally degrade bioactive gibberellins (GAs) to control plant growth, the RNS-involved genes, such as MtGA2ox10 in *M. truncatula* (Medtr4g074130), have evolved to precisely regulate gibberellic acid levels during early rhizobial infection and nodule development. Unlike other GA2ox genes, the genes in the nodule-active group are specifically upregulated in a rhizobia-dependent and nod factor-dependent manner during the early stages of nodule formation (Kim et al., 2019).

In the bHLH transcription factor superfamily (**Figure 6**) (membrane-localized basic helix-loop-helix DNA-binding transcription factor; renamed GmbHLHm1 by Chiasson et al., 2014; previously annotated as a symbiotic ammonium transporter in some genome annotations), the phylogeny identifies four legume clades. Based on the phylogenetic patterns and synteny, these four clades evidently resulted from one old segmental duplication (WGD), giving a split of clades 3 and 1 + 2; and then two old local duplications: one giving clades 1 and 2 and another giving clade 4. In the synteny plot, these local duplicates are apparent as the cluster of green-labeled genes in the center – a cluster whose age is suggested by the fact that it is present in every species except *Vitis*. There are also more recent tandem duplication nodes near the tree tips indicating recent local duplications unique to some lineages. The expression profiles reveal clear clade-level contrasts. *Chamaecrista* paralogs show nodule-biased expression in most (five of 7) cases. In contrast, within the Papilionoideae family, *Medicago* shows a strong shoot bias in clade 1, among 11 genes locally duplicated on chromosomes 4 and 6. Legume clade 3 is characterized by exclusive nodule-biased expression across all sampled species, including *Glycine* (both homoeologues of the papilionoid WGD), *Medicago* (both homoeologues), *Phaseolus* (one of two homoeologues), and *Chamaecrista*. In contrast, clade 4 shows a contrasting pattern, with *Medicago* exhibiting nodule bias, whereas *Glycine* and *Phaseolus* are more strongly expressed in other tissues, particularly shoots. These lineage-specific deviations contrast with the more uniform nodule bias across most paralogs in the *Chamaecrista* and highlight how paralogs derived from the same ancestral duplications can be rewired toward different regulatory outcomes. The results suggest that the bHLH transcription factors family first formed through ancient segmental duplications and later expanded and diversified via repeated tandem duplications. The presence of strong nodule expression in three of the legume clades (though uneven), across all four tested species, would be consistent with the involvement of ancient gamma-derived paralogs in pre-existing root or symbiosis-related functions that were later co-opted as modules into nodulation. However, it is also conceivable that the several paralogs acquired their functions in the nodule via parallel cis-regulatory evolution, for example through independent gains of transcription factor binding sites or other regulatory elements that enabled nodule-specific expression.

Genes with RNS involvement in the bHLH transcription factor superfamily show evidence of neo- or subfunctionalization, particularly regarding their recruitment to regulate nutrient transport in symbiotic structures. In soybean, GmbHLHm1 (Glyma.15G061400) is highly expressed in nodule parenchyma cells and acts to transcriptionally activate GmAMF3, an ammonium transporter, representing a specialized mechanism for regulating ammonium supply within the nodules (Chiasson et al., 2014). The key changes in MtBHLH476 appear to have been in modifications in the cis-region regulatory elements; particularly, in 12-bp RR Binding Sites (RRBS) that are targets of CRE1-dependent CRE1-dependent signaling modules (Ariel et al., 2012; Laffont et al., 2015). Other hormone-responsive elements may also be involved, as GA-responsive pyrimidine box (P-BOX) and TATC-BOX (TGGGATA) elements and auxin-responsive TGC elements (AACGAC) are present in the regulatory region of GmbHLHm1 (Ovchinnikova et al., 2023; Hu et al., 2025).

The SER6 (Serpin; serine proteinase inhibitor; LegSF.fam3.14698) superfamily (**Figure 7**) exhibits a strikingly different pattern than in the preceding three cases. In the SER6 phylogeny, tandem duplications dominate near the tip of many branches, forming dense clusters of genes from a single species. The cluster shown in *Medicago* in **Figure 7C** has 13 members, while *Chamaecrista* has seven and every other papilionoid species has at most one syntenic paralog. These tandem clusters represent more recent local expansions that occurred independently in several lineages.

The nodule-related specialization in the SER6-family genes appear to be via both copy-number expansion and regulatory changes, leading to strong, nodule-specific upregulation under drought stress and during nodule senescence (Dhanushkodi et al., 2018). Expression results show that genes from these tandem clusters often diverge in their tissue preferences, which is consistent with neo- or sub-functionalization after duplication (Innan and Kondrashov, 2010). Overall, the SER6 superfamily demonstrates that ancient WGD-derived copies provide an initial source for functional diversification, while lineage-specific tandem bursts introduce diversity and drive distinct paths of specialization.

The DNF2 (Defective in Nitrogen Fixation 2) superfamily (**Figure 8**) forms six well-supported legume clades, deriving from four segmental and two old local duplications. The local duplications are evident both in the phylogeny, in the chromosomal and gene-position information in gene IDs in clades 3 and 4 and clades 5 and 6; and also in the synteny plot (**Figure 8C**), in the three aligned columns of genes from this family. Legume clade 6 at the bottom of **Figure 8A**, shows clear nodule-biased expression in the three tested papilionoid species, derived from a retained old local duplication. The recruitment of only the leftmost of these copies (in the synteny plot, **Figure 8C**) into the nodule suggests the importance of these local duplications in the subfunctionalization and specialization of these genes in RNS. Clade 6 includes only papilionoid species plus *Cercis* and *Quillaja*, suggesting that this duplication originated in the Fabales, possibly after the split with Cannabaceae (*Parasponia* and *Trema*) and other rosid lineages (*Prunus* in our phylogeny). Notably, *Chamaecrista* is absent from clade 6, and the strongest nodule expression in *Chamaecrista* is in clade 3, where some but not all *Medicago* genes show a similar pattern, lacking in *Glycine* and *Phaseolus*.

The DNF2 gene belongs to a protein family (phosphatidylinositol phospholipase C; PI-PLCXD) that is widespread in plants, but the DNF2 gene itself has evolved a specialized, nodule-specific function. In *M. truncatula*, unlike ancestral PI-PLC genes which typically function in general signaling, DNF2 acts uniquely to suppress defense responses in the symbiosome, allowing rhizobia to persist and differentiate into nitrogen-fixing bacteroids without triggering host defense reactions (Bourcy et al., 2013; Berrabah et al., 2014).

The MtNFP/LjNFR5 and MtLIK3/LjNFR1 superfamilies (**Figures 9**, **10**) should be considered together, since genes from these families operate together in a heterodimer, and are mutually informative regarding their respective modes of evolution. The two genes together are responsible for recognizing lipochitooligosaccharide (LCO) nod factors produced by rhizobial symbionts. Although both LjNFR5 and LjNFR1 (using the gene symbols from *L. japonicus* subsequently for brevity) are receptor kinases, the domain structures and functions are different, and sequence analysis places them in distinct superfamilies. NFR5 acts as an atypical, kinase-inactive receptor necessary for initial recognition, while NFR1 acts as an active receptor kinase that initiates downstream signaling, recognizing extracellular Nod factors and transmitting a signal internally to the cell to trigger the signaling cascade that leads to nodulation (Krönauer and Radutoiu, 2021; Hansen et al., 2024).

The NFP/LjNFR5 superfamily (**Figure 9**) is relatively small and well conserved, retaining most duplications deriving from subfamily-specific WGDs within the legume family, and also showing evidence of an older segmental duplication, giving rise to two legume-family clades 1 and 2. An additional tandem duplication is evident in the non-legume species *Parasponia* and *Prunus* outgroup to clade 1. These are apparent in adjacent gene IDs in *Prunus* (Prupe.7G147400, Prupe.7G147300) and in *Parasponia* (PanWU01x14_320250, PanWU01x14_320240) in **Figure 9A** and also in the tandem duplications (adjacent green triangles for *Prunus* and *Parasponia*) in **Figure 9C**. Nodule expression is strongest in clade 1 in both papilionoid species (at least for *Phaseolus* and *Medicago*) and in *Chamaecrista*. The NFP/NFR5 gene in legume clade 1 in **Figure 9**, along with co-orthologs NFP1 and NFP2, originating via local duplication in the Rosid lineage (Finegan et al., 2025). The NFP1 and NFP2 genes are active in actinorhizal nodulation in *Discaria* and *Dryas*, and rhizobial nodulation in *Parasponia* (Finegan et al., 2025).

The MtLIK3/LjNFR1 superfamily (**Figure 10**), from the large and diverse Receptor-Like Kinase (RLK) superfamily, with Lysin-Motif (LysM) receptors responsible for recognizing specific LCO Nod factors (Zhang et al., 2009; Broghammer et al., 2012; Chiu and Paszkowski, 2020). The mode of evolution of the MtLIK3/LjNFR1 superfamily is strikingly different from that of its dimeric partner, MtNFP/LjNFR5. Using the nomenclature of Buendia et al. (2018) and Rutten et al. (2020), we focus on the LYK1-a clade (**Figure 10A**). This clade appears to have originated from the early-angiosperm WGD (Rutten et al., 2020; Liu et al., 2026), but subsequent to that, it has expanded primarily through local duplications. In **Figure 10**, there are three clades each of papilionoid, caesalpinioid, and cercidoid genes. At least two of these are the result of a tandem gene duplication that occurred around the time of the origin of the legumes (subsequent to the divergence with other Rosid species but prior to the divergence of the legume subfamilies). Additional local paralogous duplications have occurred in some genera, resulting in 11 copies on *Medicago* chromosome 5 and one copy on *Medicago* chromosome 3 (the paralogs on two chromosomes being consistent with the pre-Papilionoideae WGD).

Both the NFP/NFR5 and the LYK/NFR1 gene families show evidence of neo- or subfunctionalization to enable recognition of specific LCO Nod factors. The genes have evolved to operate as a dimeric complex, with NFR5 lacking kinase activity but acquiring the ability to bind to Nod factors, while NFR1 possesses an active kinase domain (Radutoiu et al., 2003; Buendia et al., 2016; Gough et al., 2018; Murakami et al., 2018; Müller, 2020).

### Phylogeny, expression, and synteny results for all RNS superfamilies

Phylogenies, expression plots and values, and synteny results are available for all 175 RNS superfamilies in this study in **Supplementary Data**. The superfamilies and all associated components (HMMs, sequences, alignments, trees) are available at https://data.legumeinfo.org/LEGUMES/Fabaceae/genefamilies/LegSF.fam3.W6TK/. Expression plots and associated gene trees are available as a compressed archive named “trees_and_expression_heatmaps” (https://agdatacommons.nal.usda.gov/articles/dataset/Data_from_Impacts_of_gene_duplication_in_the_evolution_of_symbiotic_root_nodule_symbiosis/30142387). Interactive synteny plots may be visualized at the Genome Context Viewer at https://www.legumeinfo.org/, or accessed from gene report pages from the Gene Search Tool.

## Discussion

Our analyses of gene families critically involved in nodulation supports the hypothesis that gene duplications of several types were important in the evolution of RNS. In particular, the γ paleohexaploidy is evident as early gene duplications, retained in most families we examined. In gene families such as NIN (**Figure 4**), the paleohexaploidy produced additional gene copies that could acquire new, specialized roles. Local gene duplications are also evident in numerous families. In gene families such as bHLH (**Figure 6**), SER6 (**Figure 7**), DNF2 (**Figure 8**), and LYK/NFR1 (**Figure 10**), local duplications have occurred around the time of the NFNC diversification, and have given rise to gene lineages that exhibit apparent RNS-related expression changes—changes that are consistent with reported functional changes.

The evolutionary model proposed by Liu et al. (2026) refines longstanding hypotheses involving co-option of several preexisting pathways—chiefly, AM symbiosis, nitrate response, and stress response—prior to the diversification of the NFNC. Methodologically, our study and Liu et al. (2026) differ in important ways. Liu et al. reconstruct the full regulatory network underlying RNS across all nodulating orders, drawing on comparative genomics and an AI-based approach to predict cis-regulatory interactions, whereas our study examines the phylogenies, synteny, and tissue-level expression of 175 individual superfamilies in detail. Nevertheless, both studies reach the same conclusion regarding the early recruitment of key genes; namely the importance of gene duplications due to the γ paleohexaploidy.

Our study adds to this picture, focusing on the dynamics and types of gene duplication in genes with RNS involvement. The subsequent elaboration or refinement of RNS may have depended on other genes, likely with some instances of independent, convergent evolution, since nodulation involving symbiont infections in fixation threads (FT-type nodulation) occurs in scattered lineages in Cucurbitales, Rosales, Fagales, and Caesalpinioideae; and nodulation involving hosting of symbionts in symbiosomes (SYM-type) occurs in the Papilionoideae and in some lineages in the Caesalpinioideae (Sprent et al., 2017; Sachs et al., 2018; de Faria et al., 2022; Liu et al., 2026).

In the exceptional case of NIN (**Figure 4**), all members of a single legume clade show strong expression in the nodule. More frequently, numerous paralogs of a given family show nodule expression; and such paralogs are frequently found in different legume clades within a superfamily, often (but not always) derived from an ancient WGD (the eudicot gamma triplication or an older WGD). The broad conservation of several superfamilies following ancient WGDs may also be interpreted in light of the dosage balance hypothesis. Genes that participate in regulatory and signaling processes are often retained after polyploidy, likely because changes in copy number can disrupt the balance among interacting components (Tasdighian et al., 2017). Although we did not directly test dosage sensitivity or network structure in this study, the persistence of conserved paralogs in several transcription factor and signaling-related families is broadly consistent with this interpretation. However, WGDs are not the only driver of gene duplication; more than a third (34.2; 6589 of 19245) of genes across the 175 superfamilies have a local paralog. Many of these local duplications are found in a small proportion of the superfamilies (such as Serpin family, **Figure 7**). A significant number of the superfamilies (13.7%; 24 of 175) have old local duplications, predating the legume diversification. Examples of such superfamilies include bHLH transcription factors (**Figure 6**) and DNF2 (**Figure 8**). Such cases are of particular interest because they provide a mechanism for subfunctionalization potentially arising in the timeframe in which nodulation arose. Further, the architecture of such genes, in clusters capable of expanding or contracting, offers a mechanism consistent with the repeated gain and loss of nodulation across the NFNC.

The variability in expression patterns observed among papilionoid species further illustrates that even closely related legumes can employ different regulatory solutions for nodulation. This is not unexpected given the diversity of nodule types within Papilionoideae (Roy et al., 2020), and highlights that homologous symbioses can nonetheless exhibit substantial divergence (Doyle et al., 2025). On a broader phylogenetic scale, Libourel et al. (2023) also demonstrated that genes initially involved in different biological processes were transcriptionally rewired into nodulation programs throughout the root-nodule nitrogen-fixing clade. This finding reinforces the idea that various regulatory solutions can emerge from a shared predisposition for RNS. In the context of the single versus multiple origins debate, this diversity sets a high bar for using expression differences as evidence against homology in more phylogenetically distant taxa (Doyle, 1994). Likewise, the diversity of infection mechanisms (e.g., infection threads versus crack entry) and the presence or absence of terminal bacteroid differentiation across legumes shows that functional variation within nodulating lineages alone does not, by itself, favor either a single- or multiple-origin scenario for RNS.

Published evidence of a predisposition-associated cis-element (PACE) in the NIN promoter within FaFaCuRo and of NIN’s NLP-inherited broad DNA-binding capacity provides a regulatory context for the early recruitment of NIN into nodulation (Shen and Feng, 2024; Doyle et al., 2025; Cathebras et al., 2026; Nosaki et al., 2026). Additionally, NIN-dependent regulation of NAD1 via conserved cis-elements links organogenesis and defense modulation (Yu et al., 2024). Our analyses did not directly test these regulatory elements, rather, we integrated these published findings with our NIN and DNF2 duplication-expression patterns to formulate a hypothesis of early recruitment followed by preservation. In particular, the presence of the PACE motif offers a mechanistic explanation for the conserved nodule expression of NIN that we observed across nodulating lineages, while the retention of nodule-active duplicates in the DNF2 family highlights how duplication and regulatory changes together may have stabilized their functions once incorporated into the symbiotic program.

The GA2ox superfamily (**Figures 5A, B**) exhibits bias shifts in the timeframe after the origin of the legume family – evident in the duplication of the papilionoid sequences in legume clade 1 in 7A. The increase in copy number in these genes, and the changes in expression, may have involved both copy-number expansion and cis-regulatory divergence in paralogs. Because the GA2ox patterns discussed here involve only papilionoid legumes, whose nodulation symbioses are homologous under both single-origin and multiple-origin frameworks, these results are best interpreted as evidence for lineage-specific diversification of homologous nodulation programs within the papilionoid lineage, including post-duplication regulatory divergence and paralogue-specific recruitment. Given these alternatives and our focus on a single superfamily, we view the GA2ox superfamily as an example that illustrates how nodulation programs diversify following gene duplication within papilionoid legumes.

Finegan et al. (2025) interpreted duplication histories in the NFP family (NFR5-like serine-threonine kinases; LegSF.fam3.03116) as evidence of convergent nodulation origins. Our findings indicate that any convergent nodulation involving this gene family would have depended on the prior angiosperm triplication (**Figure 9**). All members of the NFP family that have been shown to have nodule involvement, including genes in non-legume nodulators (at least including *Parasponia*, *Discaria*, *Dryas*, *Datisca*) are found in NFP Clade 1 in **Figure 9**. The tandem duplication is evident in the phylogeny (**Figure 9A**) and in the corresponding synteny plot (**Figure 9C**), with tandem duplications evident as local copies of genes in superfamily LegSF.fam3.03116 – apparently shared between *Parasponia* and *Prunus*. Given the apparently ancient origin of clades 1 and 2, the general conservation of duplicated genes within each clade (apart from some copy-number variation at that locus), and the strongest nodule expression being seen in clade 1, a single common origin of nodule function for the NFP-related genes appears most likely.

From gene phylogenies alone, it is not generally possible to distinguish between divergence and convergence in the origin(s) and subsequent evolution of nodule function. However, generalizing from the a wider perspective of many superfamilies of genes with SNF involvement, we see multiple evolutionary modes and potential evolutionary mechanisms involved in evolution of the complex RNS syndrome, supporting a model in which an ancestral predisposition for nodulation, potentiated by early innovations including new regulation and function for NIN, underwent subsequent modifications, with gains and losses potentially due to different critical genes in different lineages, rather than through wholesale independent gains in the various RNS lineages (Zhang et al., 2024b), consistent with evidence that much of the symbiotic program was already present in the ancestor of nodulating species (Soyano and Hayashi, 2014; Battenberg et al., 2018; Libourel et al., 2023; Doyle et al., 2025; Liu et al., 2026).

Across nine nodulation pathway stages, several genes (e.g., SIP1 and CLE-RS1/2) were not detected in our current *Chamaecrista* genome (**Figure 3**). In contrast, NCR-related orthologs are primarily documented in papilionoid species (Roy et al., 2020). Some legumes, including species of *Chamaecrista*, form nodules without symbiosomes or terminal differentiation (de Faria et al., 2022); therefore, the absence of NCR peptides in these lineages is not unexpected. These observations are consistent with subfamily-level loss or functional divergence and do not by themselves distinguish between single-origin and multiple-origin models of nodulation. However, because non-detection may reflect sampling or detection limits, HMM-guided searches, synteny interrogation, and stage-resolved expression profiling in *Chamaecrista* and its close relatives are necessary to distinguish true loss from highly divergent orthology, noting that in these studies most cases of highly divergent orthology were shown to involve pseudogenes, supporting lineage-specific loss of nodulation functions (Griesmann et al., 2018; van Velzen et al., 2019).

The backdrop of duplications includes the hypothesized pre-angiosperm zeta and epsilon, the core-eudicot gamma triplication, and lineage-specific WGDs within legumes (Jiao et al., 2011, Jiao et al., 2012; Amborella Genome Project et al., 2013; Ruprecht et al., 2017; Ren et al., 2018; Stai et al., 2019, Stai et al., 2025; Koenen et al., 2021; Zhao et al., 2021). The WGD radiation lag-time model offers an explanation for the temporal gap between the gamma triplication and the emergence of nodulation. This model posits that duplicated loci require time to accumulate coding and regulatory changes that enable complex traits (Schranz et al., 2012). In the case of RNS symbiosis, involving multiple complex modules, acquisition of necessary affiliated molecular components may have taken considerable time. The local duplications seen in key genes such as bHLH (**Figure 6**), SER6 (**Figure 7**), DNF2 (**Figure 8**), and LYK/NFR1 (**Figure 10**) provide a mechanism for such acquisitions. In each of these cases, local duplications occurred around the time of the origin of RNS, and were subsequently retained in most lineages. Local duplications also played a critical role the evolution of in LYK/NFR1. The NFP/NFR5 and LYK/NFR1 receptor pair (**Figures 9**, **10**) functions to recognize the symbiotic Nod factor and initiate the kinase transduction cascade that triggers development of the nodulation program (Krönauer and Radutoiu, 2021; Hansen et al., 2024). Subsequent expansion and contraction of genes in LYK/NFR1 responsible for recognizing specific ligands from the symbiont provide a mechanism to permit rapid evolution of host-symbiont specificity (Murakami et al., 2018; Walker et al., 2020; Yu and Zhu, 2025).

In summation, our analyses support that the novel function in the core regulator NIN was associated with an early gene duplication (likely the gamma triplication), consistent with earlier studies showing that NIN originated by duplication within the broader NLP family (Liu and Bisseling, 2020; Hsin et al., 2021; Zhao et al., 2021; Zhang et al., 2024b; Doyle et al., 2025; Liu et al., 2026). This duplication, along with cis-region regulatory changes (Cathebras et al., 2026), gave rise to the gene lineage that shows dominant expression in the nodule in all tested RNS lineages (Schauser et al., 1999; Griesmann et al., 2018; van Velzen et al., 2019). In contrast, other gene superfamilies illustrate lineage-specific modifications layered upon this shared framework. Superfamilies such as DNF2, bHLH, and LYK/NFR1, with old retained local duplications from the timeframe of the origin of RNS. Such local duplications offer a mechanism by which genes essential to RNS may have acquired new functions, in the timeframe of the origin of RNS. Expansion and contraction of local clusters of genes also provide a mechanism for potential stochastic gains and losses in various lineages, consistent with the hypothesis of multiple gains and losses of SNF across the NFNC (Kates et al., 2024). Such local duplications also help to explain the basis for distinct types of RNS in different lineages. In particular, DNF2 (and the related SymCRK pathway) is unnecessary in certain legumes that do not form infection threads (Karmakar et al., 2019). This indicates that these local duplications interact with infection mode such that different genetic solutions can produce functional nodules with or without infection threads. This synthesis supports the idea that an ancestral genetic predisposition for nodulation may have been established once, with early gene duplications playing a critical role (the gamma triplication in particular giving rise to additional copies of every gene); and that many other genes with important roles in RNS also trace their origins to gene duplications of several types – both due to ancient WGDs and to local duplications and losses of varied ages (Liu et al., 2026). Expression changes in the superfamilies that we examined are seldom simple or exclusive to a single gene lineage. Rather, expression is more often present across tissues and shared across several paralogs. If a nodule is a parade, the many participants in the parade would not be expected to have joined simultaneously, because the developmental and metabolic modules that comprise nodulation (e.g., lateral root morphogenesis, mycorrhizal signaling, tip growth, nitrogen metabolism) are likely to have distinct evolutionary histories.

## Data Availability

All scripts used for RNA-seq processing, expression summarization, and downstream analyses are available at the following GitHub repository: https://github.com/hyun52/RNS_gene_expression_analysis.

## Supplementary material

Supplementary Table 1 provides the TBLASTN results of five RNS-related proteins (CLE-RS1/2, SIP1, DNF4/NCR211, DNF7/NCR169, and SNARP2) against the *Chamaecrista fasciculata* genome assembly.

Supplementary Table 2 provides the full matrix of pairwise Wilcoxon rank-sum tests with Benjamini–Hochberg correction, assessing differences in per-superfamily gene counts across all species.

Supplementary Figure 1 shows the distribution of legume clade counts per superfamily.

Supplementary Figure 2 presents the principal component analysis (PCA) of gene expression bias patterns at the superfamily level across four legume species (*Chamaecrista, Glycine, Medicago*, and *Phaseolus*), illustrating overall structure and divergence of tissue-specific expression biases.

Supplementary Figure 3 summarizes the classification and frequency of tissue-specific expression bias categories associated with nodulation across legume clades.

Supplementary Figure 4 is the phylogeny of the NIN superfamily (LegSF.fam3.02933) across legumes and outgroup species, with bootstrap support values. This figure corresponds with **Figure 4** in the main document.

Supplementary Figure 5 is the phylogeny of the GA2ox superfamily (LegSF.fam3.00582) across legumes and outgroup species, with bootstrap support values. This figure corresponds with **Figure 5** in the main document.

Supplementary Figure 6 is the phylogeny of the bHLH transcription factors superfamily (LegSF.fam3.02030) across legumes and outgroup species, with bootstrap support values. This figure corresponds with **Figure 6** in the main document.

Supplementary Figure 7 is the phylogeny of the SER6 superfamily (LegSF.fam3.14698) across legumes and outgroup species, with bootstrap support values. This figure corresponds with **Figure 7** in the main document.

Supplementary Figure 8 is the phylogeny of the DNF2 superfamily (LegSF.fam3.00681) across legumes and outgroup species, with bootstrap support values. This figure corresponds with **Figure 8** in the main document.

Supplementary Figure 9 is the phylogeny of the NFP superfamily (LegSF.fam3.03116) across legumes and outgroup species, with bootstrap support values. This figure corresponds with **Figure 9** in the main document.

Supplementary Data Sheet 1 (rns_families_counts_2025-09-19.xlsx) containing superfamily names, counts, species composition, clade assignments, and expression matrices.

Supplementary Data Sheet 2 (rns_local_dups_2025-09-19.xlsx) summarizing paralog counts, genomic positions, and ancient local duplications.

Supplementary Data Sheet 3 (rns_pairwise_bias_results_09-19.xlsx) reporting pairwise expression bias results and summary counts across legume clades and superfamilies.
