# Supplementary figures and images for: Impacts of gene duplication in the evolution of symbiotic root nodule symbiosis in legumes
Source: Front Plant Sci. 2026 May 12;17:1784647. doi: 10.3389/fpls.2026.1784647 (PMC13224818; doi:10.3389/fpls.2026.1784647)

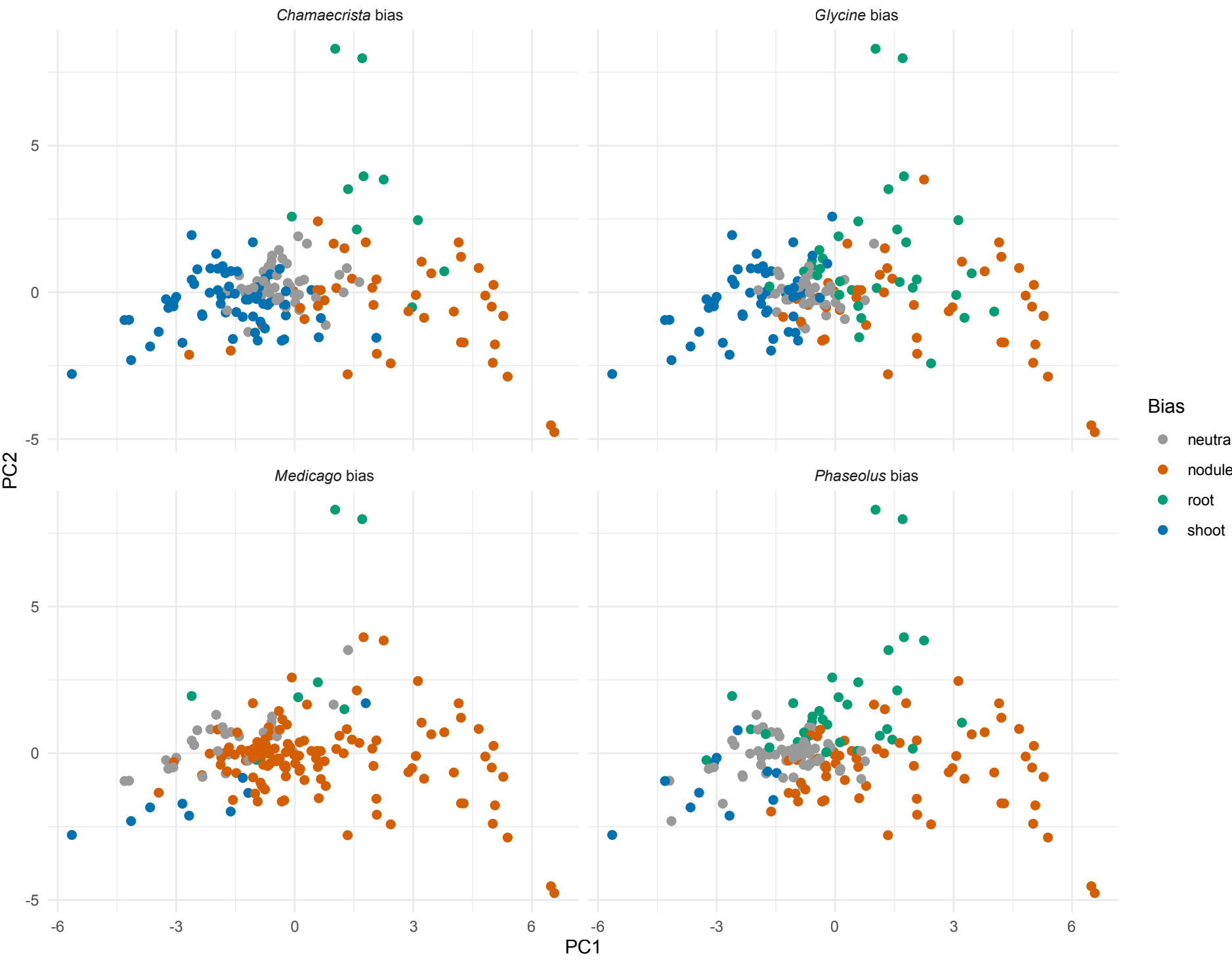

Supplement: Supplementary file 1 [file DataSheet2.pdf]

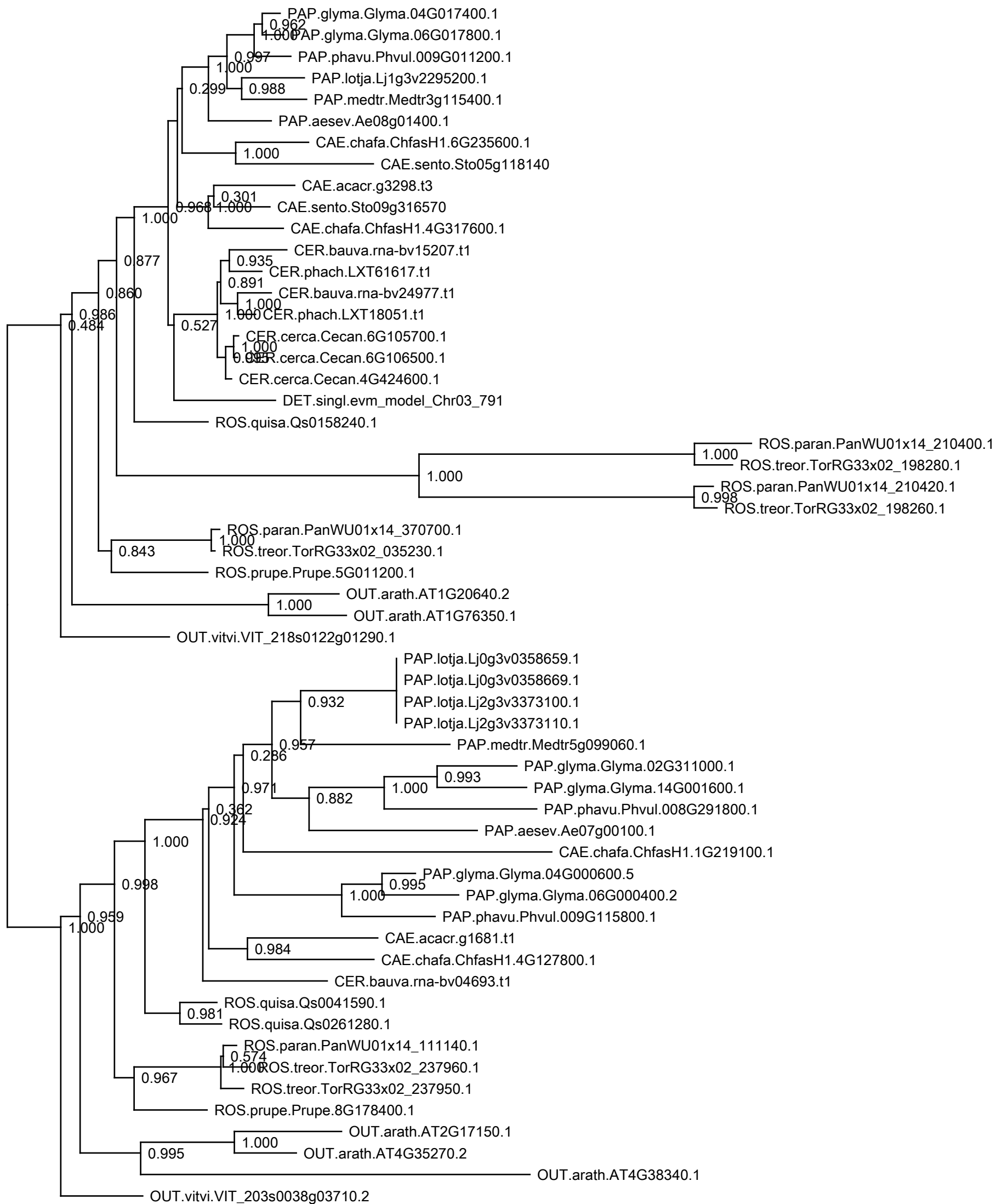

Supplement: Supplementary file 3 [file DataSheet4.pdf]

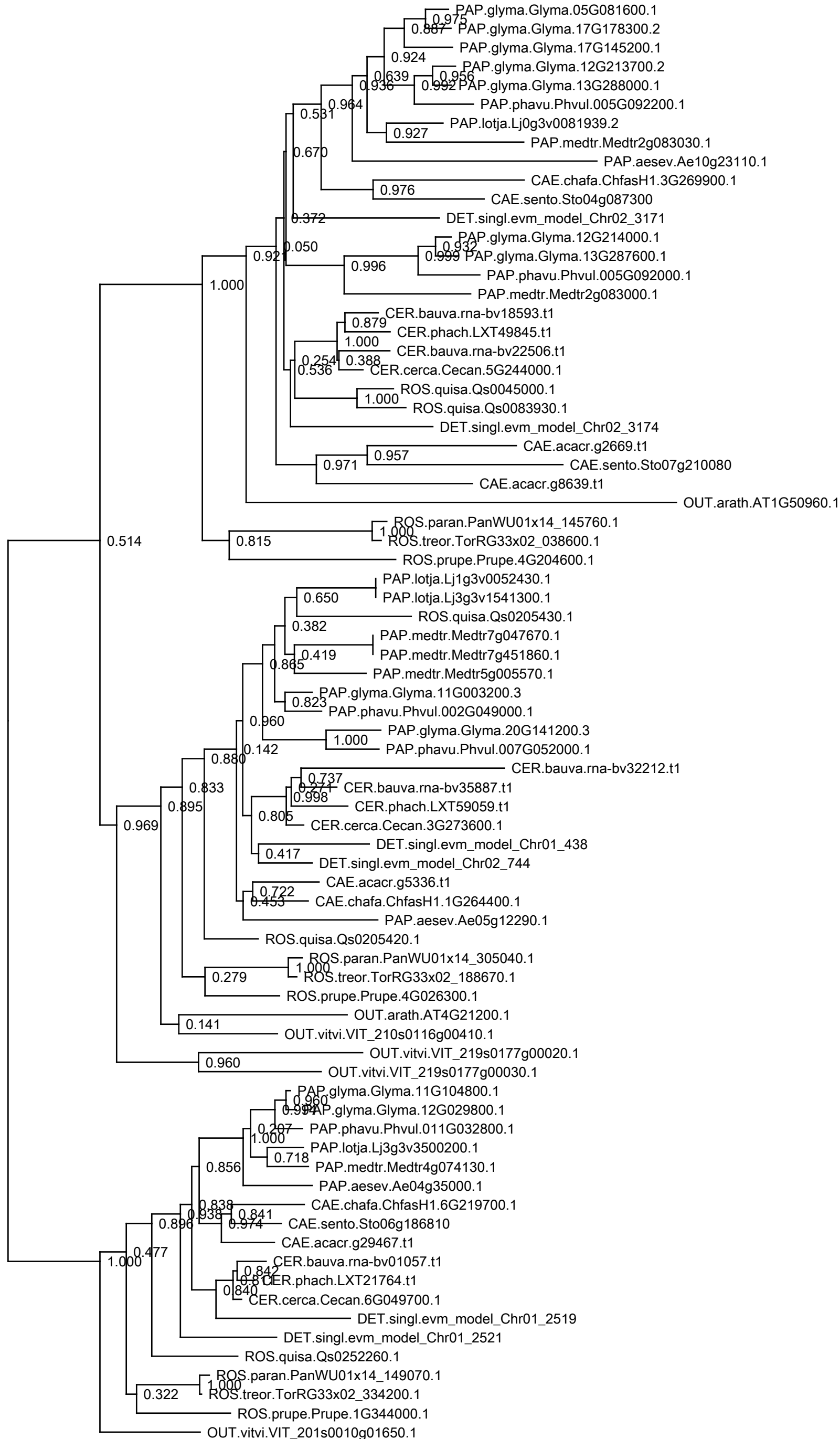

Supplement: Supplementary file 4 [file DataSheet5.pdf]

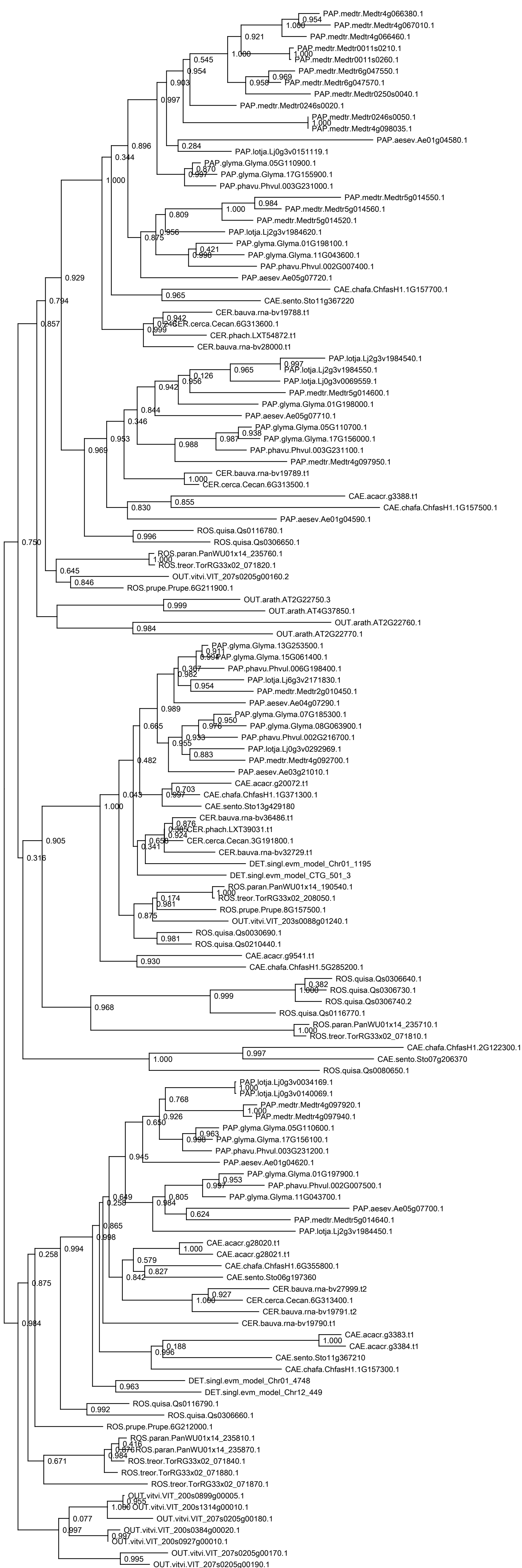

Supplement: Supplementary file 5 [file DataSheet6.pdf]

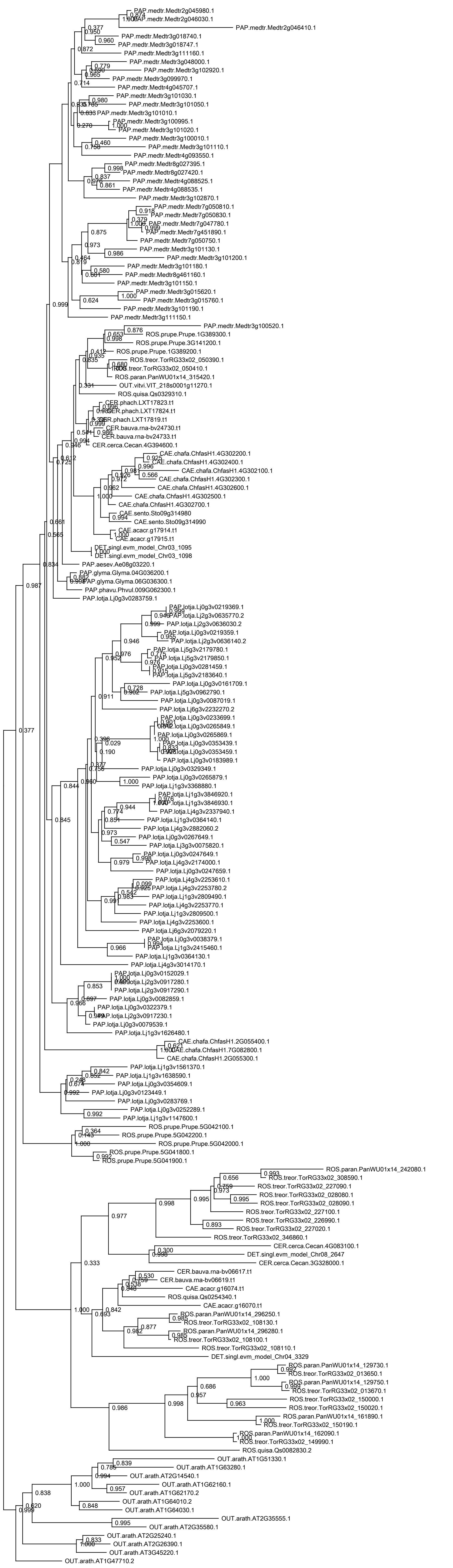

Supplement: Supplementary file 6 [file DataSheet7.pdf]

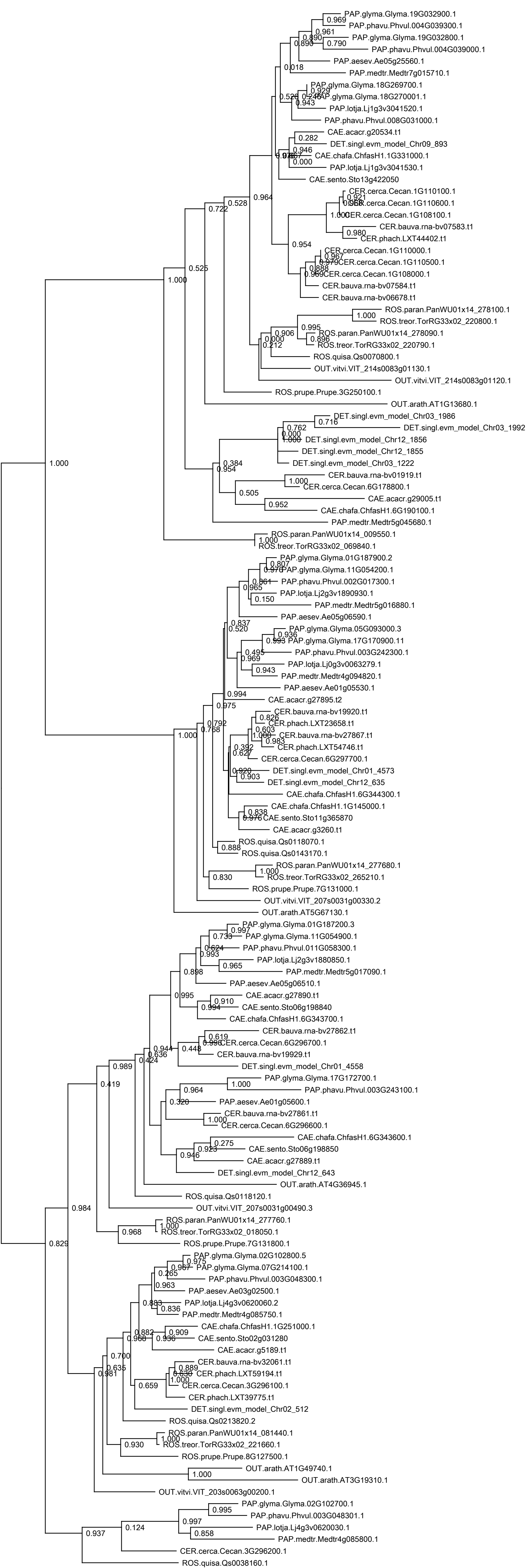

Supplement: Supplementary file 7 [file DataSheet8.pdf]

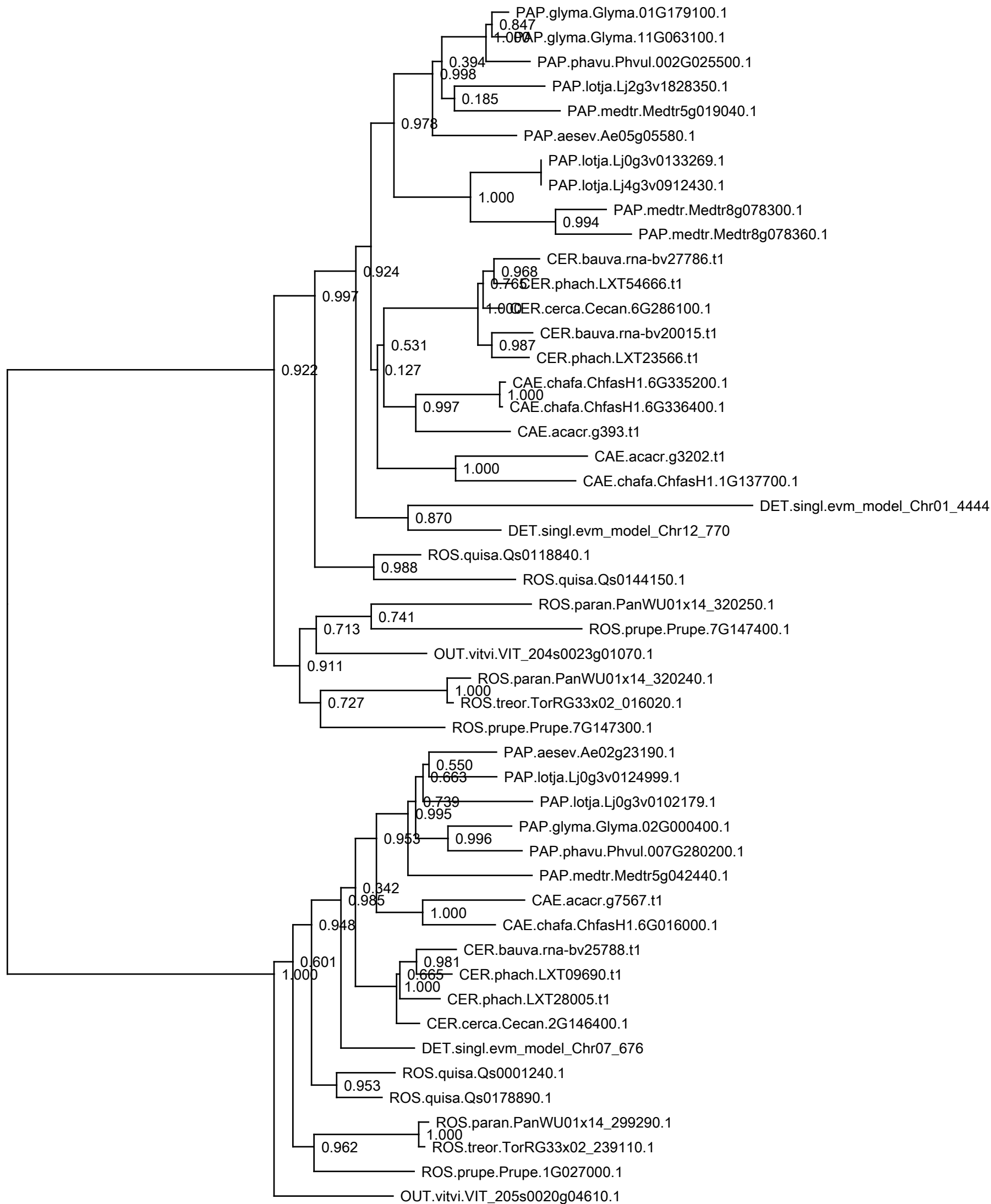

Supplement: Supplementary file 8 [file DataSheet9.pdf]

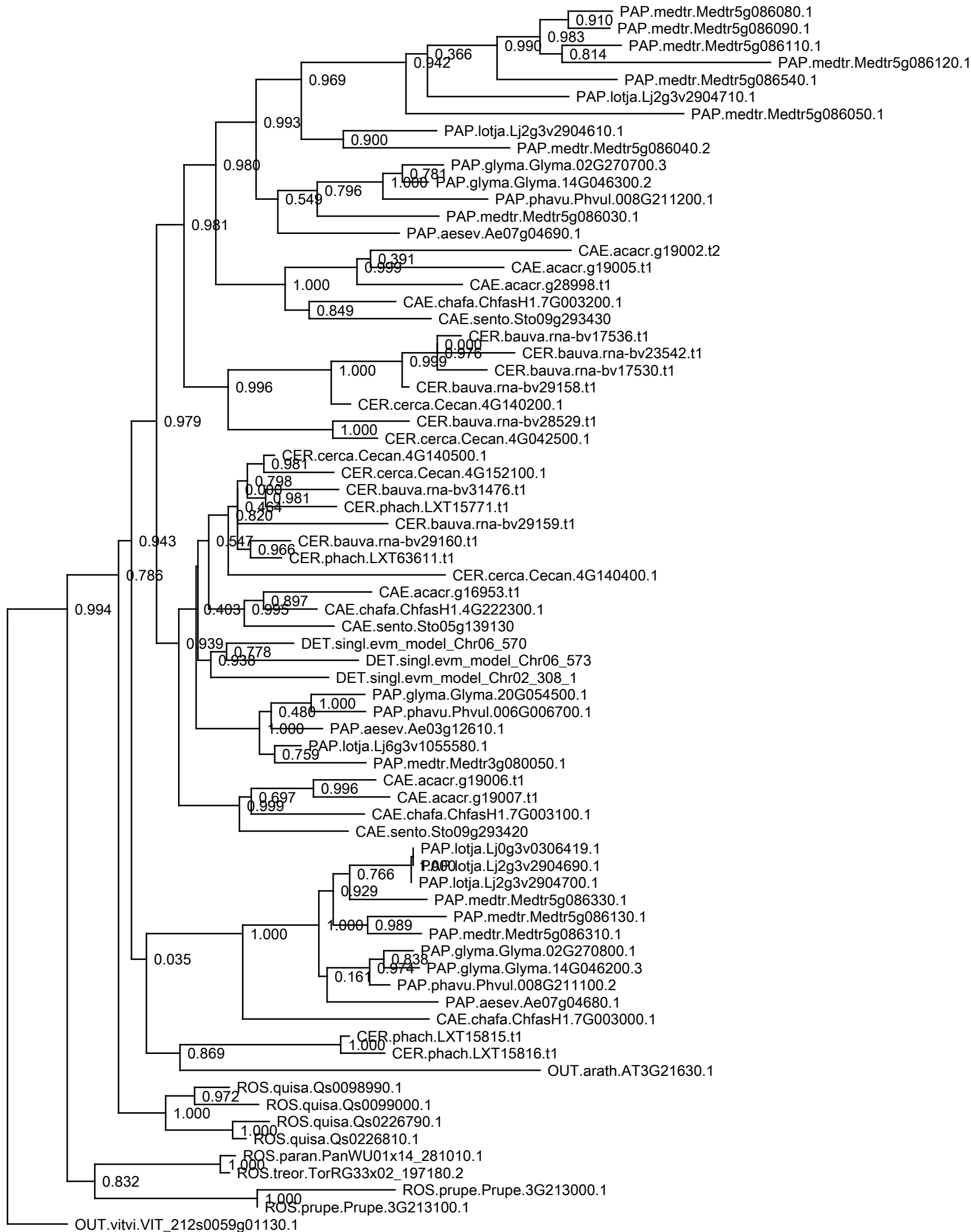

Supplement: Supplementary file 9 [file DataSheet10.pdf]

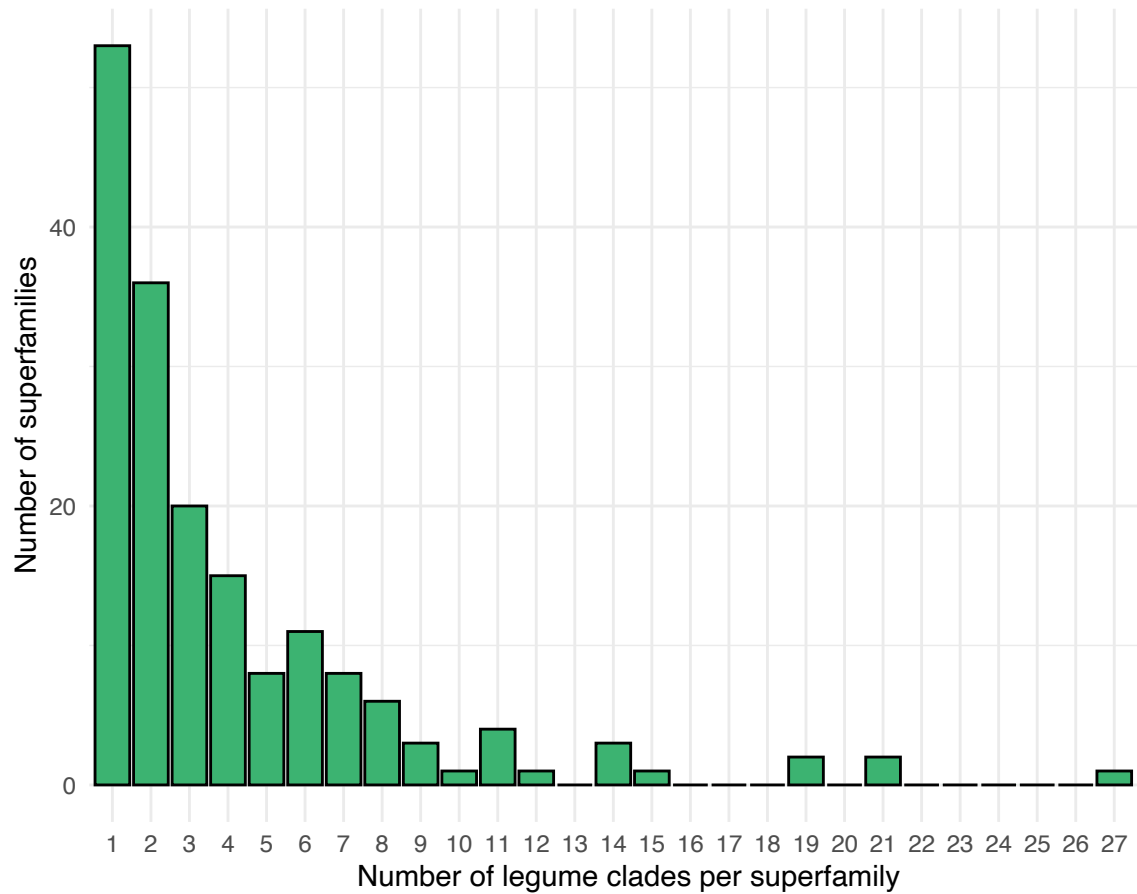

Supplement: Supplementary Figure 1 — shows the distribution of legume clade counts per superfamily. [file DataSheet1.pdf]
