# Supplementary material for: Impacts of gene duplication in the evolution of symbiotic root nodule symbiosis in legumes
Source: Front Plant Sci. 2026 May 12;17:1784647. doi: 10.3389/fpls.2026.1784647 (PMC13224818; doi:10.3389/fpls.2026.1784647)
Supplement: Supplementary file 2 [file DataSheet3.pdf]

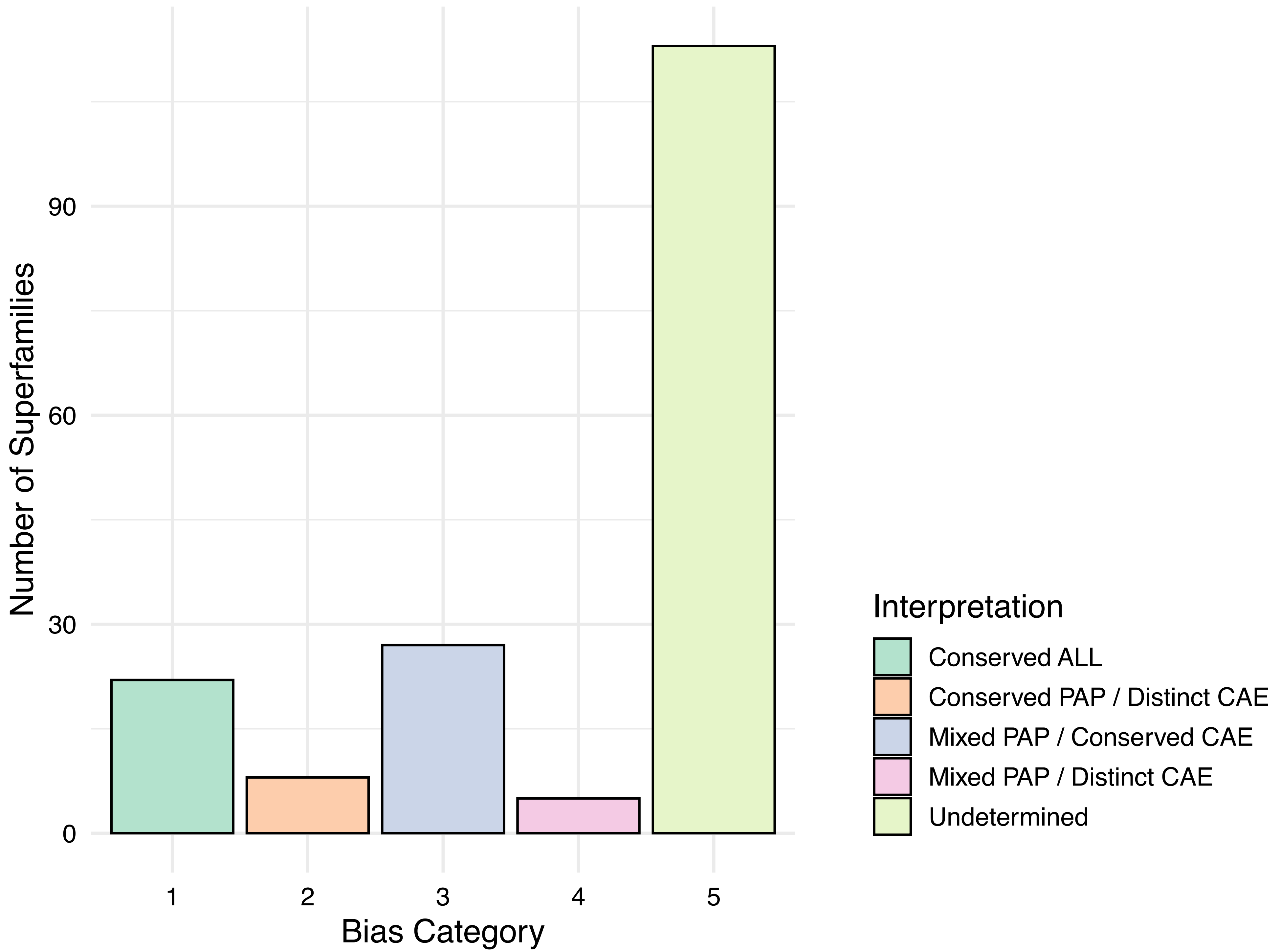

Number of Superfamilies
0
30
60
90
1
2
3
4
5
Bias Category
Interpretation
Conserved ALL
Conserved PAP / Distinct CAE
Mixed PAP / Conserved CAE
Mixed PAP / Distinct CAE
Undetermined
